# COVID-19 resilience index in European Union countries based on their risk and readiness scale

**DOI:** 10.1371/journal.pone.0289615

**Published:** 2023-08-04

**Authors:** Somaya Aboelnaga, Katarzyna Czech, Michał Wielechowski, Pavel Kotyza, Lubos Smutka, Kennedy Ndue

**Affiliations:** 1 Department of Urban Regional Development, Faculty of Urban and Regional Planning, Cairo University, Giza, Egypt; 2 Department of Econometrics and Statistics, Institute of Economics and Finance, Warsaw University of Life Sciences, Warszawa, Poland; 3 Department of Economics and Economic Policy, Institute of Economics and Finance, Warsaw University of Life Sciences, Warszawa, Poland; 4 Department of Economics, The Czech University of Life Sciences, Prague, Czechia; 5 Department of Trade and Finance, The Czech University of Life Sciences, Prague, Czechia; 6 Institute of Agricultural Economics, Budapest, Hungary; Alexandru Ioan Cuza University: Universitatea Alexandru Ioan Cuza, ROMANIA

## Abstract

Addressing risks and pandemics at a country level is a complex task that requires transdisciplinary approaches. The paper aims to identify groups of the European Union countries characterized by a similar COVID-19 Resilience Index (CRI). Developed in the paper CRI index reflects the countries’ COVID-19 risk and their readiness for a crisis situation, including a pandemic. Moreover, the study detects the factors that significantly differentiate the distinguished groups. According to our research, Bulgaria, Hungary, Malta, and Poland have the lowest COVID-19 Resilience Index score, with Croatia, Greece, Czechia, and Slovakia following close. At the same time, Ireland and Scandinavian countries occupy the top of the leader board, followed by Luxemburg. The Kruskal-Wallis test results indicate four COVID-19 risk indicators that significantly differentiate the countries in the first year of the COVID-19 pandemic. Among the significant factors are not only COVID-19-related factors, i.e., the changes in residential human mobility, the stringency of anti-COVID-19 policy, but also strictly environmental factors, namely pollution and material footprint. It indicates that the most critical global environmental issues might be crucial in the phase of a future pandemic. Moreover, we detect eight readiness factors that significantly differentiate the analysed country groups. Among the significant factors are the economic indicators such as GDP per capita and labour markets, the governance indicators such as Rule of Law, Access to Information, Implementation and Adaptability measures, and social indicators such as Tertiary Attainment and Research, Innovation, and Infrastructure.

## Introduction

Corona Virus Disease (COVID-19) went down to history globally as one of the global shocks that caught the world unaware. The disease had severe ravaging effects globally, cutting across all sectors [1]. The impacts and effects of the disease were felt across all divides of economic development, spurring debate on the level of global preparedness in tackling such threats. This necessitates concerted efforts where researchers, policymakers, and other stakeholders pool resources to ensure they establish systems that will withstand such shocks in future (resilient) systems. However, the level of preparedness and acceptability of such a move has not been much welcome by different actors as different countries exhibit different levels and have diverse mechanisms. This necessitates a common ground rule to ensure that no actor setting up such an initiative feels short-changed. To achieve this, this study delves into in-depth research to investigate the level of preparedness among the European Union member states by developing a COVID-19 Resilience Index (CRI). Establishing such a tool is a plausible pathway toward implementing the Just Transition mechanism, where the Union can develop a diagnostic tool to determine which country requires intervention in what aspect and where other countries can benchmark from. Despite EUs, level of development as an advanced economy, COVID-19 exposed significant fault lines and fragilities in current systems forcing us to consider possibilities for post-pandemic development rethinking and transformations [2, 3]. The impact of the COVID-19 pandemic on the global world economy and global health has been significant [4]. Globally, governments had to react, revamping their existing systems to stop or slow down the transmission of the disease [5]. All countries were at risk and needed to prepare for and respond to the COVID-19 pandemic [6].

Risks associated with pandemics are typically managed through reactive approaches, which include researching more information about the severity of the pandemic during its existence and implementing appropriate strategies accordingly. However, efficiency in the process at the national level would be more effective by devising proactive strategies to minimise the impact of such pandemics [7]. Contrarily, pandemics are considered very low-probability catastrophic and irreversible events linked to deep uncertainty about their timing and severity [8]. Additionally, decision-makers at all tiers of the government have limited options for implementing proactive measures during the pandemic [9].

Although a high level of research on combating the disease has taken place to establish control mechanisms, determining the country’s readiness to cope with the pandemic threats is not easy, and research on it remains scanty. It varies from assessing performance in the reduction of COVID-19-related mortality, supporting vaccination programs to constrain future pandemic threats and supporting the recovery of socio-economic systems [10, 11]. Independent Panel for Pandemic Preparedness and Response recommends preparing the world for the future so that the next disease outbreak does not become a pandemic. The main question is how such resilient systems can be measured and evaluated [12]. Establishing standalone systems alone is not enough, as development is a relative process. This necessitates the development of a common framework that can be applied across all countries and regions or even cascade downwards to the village level.

We conduct an in-depth study of all the EU countries to evaluate their readiness to handle risks concerning the pandemic scenario. This is achieved by identifying groups of the European Union countries and grouping them. This is further subjected to classification whereby MS are grouped based on their preparedness nature characterised by a similar CRI. This is realised by developing a CRI index reflecting the countries’ COVID-19 risk and their readiness for a crisis, including a pandemic. Finally, we apply the matrix method to assess the correlation between risk and readiness to scale countries’ COVID-19 resilience by adopting the methodology developed by the University of Notre Dame. However, there is little or no information about the context. Existing research has overemphasised countries’ readiness for pandemics or risks related to such crises. Our contribution is that we first use matrix analysis to develop countries’ COVID-19 resilience index that combines both readiness and risk factors. It allows for obtaining more complex and exploitable results. We conduct the study for all the EU countries. Our study could create a new perspective for discussing similarities and differences in crisis readiness (at risk) among the EU member countries. We believe that the self-developed COVID-19 Resilience Index might constitute a valuable tool to assess a country’s risk and readiness for future crisis situations, including pandemics. The developed CRI is meant to help the decision-makers either improve the country’s status or tackle their risk by implementing the right policies targeting sustainable development under different circumstances and crises.

This paper comprises four sections, the next being a literature review of the country’s COVID-19 risks and readiness factors. Finally, the posterior section sets out there and describes the material and research methods and strategies used, followed by the empirical findings and analysis and the conclusions.

## Literature review

There are several complex factors that need to be taken into account when assessing and enhancing a country’s resilience and preparedness to combat a major pandemic threat. We provide a summary of the literature regarding indicators that can be categorised as risk indicators and measurements of a country’s readiness for a pandemic.

### Risk factors

Tackling any risk is a subset of clearly understanding it. A risk can be defined in its simplest form as the potential losses from a particular hazard to a specified risk element in a given future period [13]. Risks are further defined and schematised mathematically as the product of three determinants, i.e., hazard, vulnerability, and exposure [14, 15]. Pandemics fall under natural disasters, with the COVID-19’s hazard being its potential to occur in the future, while its exposure is the total population in the given area subjected to its future occurrences [16]. Additionally, their vulnerability is the propensity of exposed elements, i.e., human beings, to suffer adverse effects when impacted by pandemic events [15].

#### Hazard indicators

A fundamental variable expressing the COVID-19 hazard is the country’s relative number of COVID-19 cases. However, applying mathematical models and reporting the number of COVID-19 cases belongs to the risk factors affecting domestic economies [17]. Therefore, the greater the number of COVID-19 cases, the higher risk of future infectious diseases contracting. However, this is linked to population immunity, including herd immunity, i.e., retaining the surviving specimens and not introducing new ones from a different herd [18]. The World Health Organisation (WHO) and the global scientific community apply the herd or population immunity concept to the human population, including the number of vaccinated individuals next to those who have survived the disease [19]. The concept of population immunity shifts to the discussion on the required threshold of the vaccinated population required to achieve population (herd) immunity [19–21].

Herd immunity is achieved by protecting people from the virus, not by exposing them to it [6]. In Layman language can be translated that, besides factors such as COVID-19 new cases/deaths or degree of vaccination, the changes in human mobility might constitute an important variable that expresses the COVID-19 hazard. Sigler *et al*. claim that human mobility should be considered the key indicator of the spread of the pandemic [22]. Human mobility’s impact on the COVID-19 spread has been analysed by many researchers, including [23–26].

#### Vulnerability indicators

Vulnerability factors refer to the propensity of people to suffer adverse effects when impacted by the COVID-19 pandemic, including population density, diet habits, urbanisation, environmental issues, and government policy [27].

As SARS-COV-2 transmits between humans via direct contact [28], the common perception is that the novel coronavirus spread should positively correlate with population density [27, 29–31]. Ilardi *et al*. [32] find that population density positively correlates with COVID-19 morbidity and mortality. In contrast, Carozzi *et al*. [33] reveal that density affects the timing of the COVID-19 outbreak in each county, with denser locations more likely to have an early outbreak. However, he does not observe any linkage between population density and COVID-19 cases and COVID-19-related deaths.

Research points to a possible nexus between the urbanisation process and the threat of disasters [34–36]. Shekhar *et al*. [37] reveal that the leading urban centres are highly susceptible to global risks, including infectious diseases. Moreover, many risk drivers are linked to the urbanisation process, which can increase the total exposure and vulnerability of the nation in which they are. For example, urban air pollutants might increase COVID-19 case mortality rates, as indicated by [38]. However, urban residents are more concerned about the COVID-19 virus than rural residents [39]. Therefore, underreporting COVID-19 cases could lead to a false sense of security among rural populations. Expected lower case counts in rural areas due to lower population density are unfortunately associated with greater potential COVID-19 risk among the older population and limited access to medical care. They cause significant disparities in COVID-19 mortality [40]. Overall, the literature review implies that urban concentration increases COVID-19 vulnerability; however, it is not so obvious.

WHO has announced dietary guidelines during the COVID-19 outbreak, highlighting the importance of balanced nutrition to maintain a robust immune system and prevent or minimise chronic diseases and infections [41]. However, based on Bousquet *et al*. [42], the diet represents only one of the possible causes of the COVID-19 epidemic, and its importance needs to be better assessed.

Sustainability anxiety is expanding globally [43–45]. Thus, environmental issues are the critical determinants of a vulnerability to a crisis such as a pandemic. The EEA considers COVID-19 as a late lesson from an early warning. Environmental degradation increases the risk of pandemics [46]. COVID-19 emerged and escalated through the complex interplay between drivers of change, such as ecosystem disturbance, urbanisation, international travel, and climate change. Earlier studies indicated that environmental factors could also play a role in the dynamics of COVID-19 transmission [47]. Maheswari *et al*. reveal that air pollution enhances susceptibility to COVID–19 disease [48]. Air pollution affects the spread and increase of COVID morbidity and mortality [49]. Higher air pollution increases COVID-19 mortality [50]. Coccia finds that the health effects of air pollution exposure can extend beyond cardiopulmonary systems, accelerating the diffusion of future pandemics similar to COVID-19 [51].

The rapidly increasing numbers of COVID-19 infections and deaths induced national governments to implement various restrictions and lockdowns [52, 53]. Extensive empirical research reveals the significant impact of the stringency of anti-COVID-19 policy on the epidemic status [54–57]. McGrail *et al*. show that implementing social policies reduced the novel coronavirus spread rate worldwide [58]. The empirical results show that government response stringency significantly negatively impacts the number of COVID-19 confirmed cases [59]. Jinjarak *et al*. reveal that more stringent pandemic policies were associated with lower mortality rates [60]. Hence, we can assume that the stringency of the anti-COVID-19 policy decreases the vulnerability of the pandemic spread.

#### Exposure indicators

Exposure factors reflect society’s sensitivity to the more severe course of COVID-19, i.e., they are related to the age and health of people. According to [61, 62], advanced age and obesity are key factors associated with the increased COVID-19 spread and overall mortality. Generally, individuals over 65 years are more vulnerable to the COVID-19 virus than young people [54]. Rashedi *et al*. finds that ageing negatively impacts lung function and delays the activation of the acquired immune system [63]. The virus can become more reproducible, generating more pro-inflammatory responses and increasing the risk of death. Pizano-Escalante *et al*. show that frailty and COVID-19 harmed seniors [64]. Rod *et al*. find that diabetes might be the most consistent comorbidity predicting COVID-19 disease severity [65]. Peric and Stuling indicate that diabetes mellitus predisposes to a severe episode of COVID-19 and doubles the risk of dying from lung disease and cardiac disease [66]. Most research studies confirm that diabetes belongs to the COVID-19 risk factors and contributes to the severity and mortality of patients with this disease [67].

In the paper, apart from COVID-19 risk factors, we also consider factors related to a country’s readiness for any crisis situation, particularly a pandemic. Well-known readiness factors are linked to economic, governance, and social policy [68].

### Readiness factors

The preparedness and prevention of pandemic events should be integral to the country’s development process. The fundamental variable that illustrates the country’s readiness for a crisis is the country’s economic stance, measured, for example, by GDP per capita or GDP growth rate. [69, 70], among others, mention GDP per capita as a vital factor in a country’s readiness for the COVID-19 pandemic. The greater GDP per capita, the higher the likeliness of the country’s readiness.

#### Economic indicators

Labour market stance and fiscal situation are other critical economic determinants of a country’s readiness for adverse effects of external crisis shocks. Gavriluță et al. [71] examine the COVID-19 pandemic’s impact on the labour market. They discover a direct link between gender (women) and lower employability rates in the EU-28. These empirical findings offer valuable insights into the relationship between employability and sustainability. David and Pienknagura [72] found that in countries where informality is commonplace, where a small share of employment can be achieved remotely, and where government effectiveness is weak, there are smaller declines in cases after lockdown measures are tightened relative to other countries. It is linked to the fact that, in these countries, mobility decreases less after containment policies are implemented, thereby facilitating the spread of the disease. In addition, social workers might contribute to promoting public and community health during the initial phase of COVID-19 [73]. Afonso and Hauptmeier prove the responsiveness of primary balances to the government’s indebtedness [74]. The country’s fiscal situation, including primary balance and public indebtedness, shapes public spending [75]. However, fiscal rules constrain budget-makers, cutting much of their authority to decide revenue and spending policy [76]. Klose and Tillmann [77] claim that domestic fiscal policy, the macroprudential policy as well as monetary policy support the countries’ recovery from the crisis. Moreover, flexible fiscal rules include features to accommodate exogenous shocks, e.g., COVID-19 [78, 79]. Hochrainer-Stigler [80] observes an increase in fiscal risk against natural disasters due to the COVID-19 pandemic. Moreover, he reveals that the novel coronavirus pandemic has more substantially hit poor and climate-vulnerable economies, while the more developed countries tend to be more COVID-19 resilient. Nevertheless, Rawdanowicz *et al*. [81] claim that high debt levels in developed countries make public finances vulnerable to future adverse shocks. The above-mentioned studies indicate that an excellent fiscal stance, including a positive primary balance, increases the country’s readiness for any crisis situation.

#### Governance indicators

Governance policies are another crucial determinant of a country’s readiness for a crisis situation. Kahn [82], analyzing the effects of governance on disaster fatalities and damages, observes that democratic countries outperform other forms of governance, as democratic governments implement anti-disaster measures to mitigate the negative consequences of hazards. Raschky [83] confirms that institutional framework is a critical determinant of vulnerability and resilience to disasters. The willingness of the public to comply with the proposed government restrictions is crucial for controlling the spread of COVID-19 [84]. The health crises we experienced in the past have confirmed that trust in the government significantly increases the chances of society to overcome the crisis [85]. Although Chisadza *et al*. [86] present a non-linear association between government effectiveness and the number of deaths in the different economic classifications of countries, Petrovic *et al*. view trust in government as an important factor that affects countries’ readiness for crisis periods [87]. Bargain and Aminjonov [88] show that the effect of anti-COVID-19 policy stringency is more pronounced in high-trust regions. One important aspect to explore in future research is vaccine hesitancy among the population, as it can significantly impact a nation’s performance (Verger and Peretti-Watel [89]). The success of COVID-19 vaccination efforts in countries is closely linked to the level of public trust, which needs to be established and strengthened for effective pandemic crisis management (Soveri et al., [90]). Furthermore, Eurohealth [91] demonstrate that countries with efficient vaccination plans tend to have higher levels of governance indicators, such as Government Effectiveness, Regulatory Quality, and Rule of Law, compared to countries with less effective vaccination rollouts.

During the COVID-19 pandemic, access to information constitutes a significant issue. It mainly concerns the mass media. González-Padilla and Tortolero-Blanco [92] claim that social media has the great benefit of delivering educational content quickly during the COVID-19 era, e.g., Chan *et al*. [93] developed an infographic on airway management in suspected or confirmed COVID-19 patients.

At the time of the crisis, adaptability issues seem crucial both for the entire country and its elements. Organisations are in danger of experiencing unimaginable disruptions. The leader’s primary objective would be to reopen, recover the business, and begin to manage a crisis. Consequently, employees can be most at risk in several ways [94]. Financial risks, including credit, liquidity, and operational risks, are some more prevalent and unique financial concerns of firms [95], particularly in economic instability and uncertainty. Moreover, overconfidence is an empirically confirmed cognitive bias that negatively affects economic outcomes [96].

#### Social indicators

Besides economic and political factors, a country’s readiness for a crisis is linked to social issues, including inequality, education, research and innovation, and health. Pickett and Wilkinson [97] consider income inequality as a subset of social disparity in health and mortality. However, [98, 99] observed increased COVID-19 transmission and mortality in the poorest countries due partly to overcrowded housing and working conditions. Moreover, education and education programmes help prepare the country for any crisis. [100] emphasise the importance of effective public health education to reduce daily and cumulative COVID-19 mortality. Czech *et al*. find that a higher rate of tertiary education increases mobility changes during COVID-19, while the greatest response of human mobility reaction to COVID-19 refers to the most developed countries [101]. Additionally, the greater the tertiary education rate is, the greater the rules of obedience, including handwashing, which reduces the total and newly confirmed COVID-19 cases [102].

Regarding pre-COVID-19 readiness, Ramalingam, Prabhu, and Caballero-Morales [103, 104] claim that knowledge and innovation matter. Furthermore, Lv *et al*. [105] observe that the COVID-19 pandemic has intensified the need for government and urban search for and use of more innovative and safer technologies, including intelligent transportation, to prevent future epidemics. In addition, more open innovation strategies can help businesses to compete against the COVID-19-induced market downturn [106, 107].

Lastly, access to and quality of healthcare represent a fundamental issue regarding the country’s preparedness for the pandemic. Publicly funded healthcare is a substantial part of government spending in most developed countries, including the EU member states [108]. WHO [109] claims that public health and social measures (PHSMs) are a key strategy to reduce the transmission of pathogens with epidemic or pandemic potential. These include non-pharmaceutical interventions that can be taken by individuals, institutions, communities, local and national governments, and international bodies to slow or stop the spread of infectious diseases, such as COVID-19.

### Countries’ risk and readiness for a pandemic: Nexus between the analysed factors

Pandemics, regardless of their nature, affect society’s economic, environmental, and social lifestyle performance. This influences the ability of the present society to focus on its long-term development [110]. The imbalance between society’s economic, environmental, and social fabric underpins development trajectories in any fight against the pandemic. Although these processes are amplified by the current economic system that overvalues private goods, undervalues the common good, and has not given much thought about what the goals of economic growth should be in times when income, wealth, and opportunity inequalities have been increasing rapidly, and the effects of climate change become increasingly more visible to see coupled with the pandemic effects worsens the situation in Europe.

Therefore, forging a resilient economy creates an opportunity to create buffer zones for future risk, thus reducing unsustainable trends. Although economic indicators are insufficient to address sustainability, promoting multistakeholder engagement and a bottom approach when implementing these economic practices to ensure the local communities voices feature in the discussion can increase the resilience of communities in the emergence of pandemics. Additionally, the bottom-up approach ensures local values and broader community engagement is prioritised where issues besides economic growth can even be addressed based on the local experience [111].

However, it must be when dealing with establishing resilient systems to pandemics, and it is a shock absorber against shocks or irreversibilities, which can harm the path towards attaining sustainable development. Thus a multi-actor approach to ensure any weak link of the system can be detected at earlier stages is fundamental to ensure these impacts do not exert pressure on the system’s sustainability.

Noy et al. [112], measuring the economic risk of epidemics worldwide, observe that agricultural areas and high numbers of younger populations are linked to higher country vulnerability, while countries with higher geographic, social, and cultural disparity, receiving more overseas incomes, are more resilient. Diop et al. [113], constructing COVID-19 economic vulnerability and resilience indices for 150 countries worldwide, and four principal world regions, observe that on average European counties are the least vulnerable economically and most resilient economically to COVID-19. Marti and Puertas [114], analysing European countries’ vulnerability to the COVID-19 pandemic, show that Eastern European countries should allocate their resources to tackle health and societal challenges. On the other hand, countries possessing a higher GDP per capita and those that have experienced severe impacts from the coronavirus will need to implement modifications in their employment structures to curtail the adverse consequences. Coccia [51] creates indexes of resilience and preparedness to measure the performance of EU countries to face pandemic threats. He reveals that each nation possesses certain vulnerabilities, with none exhibiting high preparedness to tackle a major epidemic or pandemic. Further, his findings imply that countries with smaller population size and/or superior public governance, coupled with significant health system expenditure, fared better during the COVID-19 pandemic crisis.

According to Rai et al. [115], it is widely acknowledged that the COVID-19 pandemic is likely to have long-lasting and significant impacts on socioeconomic systems, food security, and livelihoods. The authors argue that in order to address these challenges, policymakers should prioritize the establishment and maintenance of a robust healthcare system, promote environmental sustainability, and encourage the adoption of a circular economy. By viewing the various effects of COVID-19 through the lens of Sustainable Development Goals (SDGs) and their relationship with sustainability and nexus indicators, a comprehensive understanding of the pandemic’s implications can be achieved. Conducting a pragmatic assessment of COVID-19’s consequences can enhance our understanding of vulnerability, preparedness, and potential strategies for sustainable management. COVID-19 has derailed progress toward SDGs, as all SDGs are interlinked; health systems and the health and well-being of the population are directly affected by the pandemic while impacts on prosperity, education, planetary health, and food insecurity are indirect due to pandemic responses. SDGs reports have shown uneven progress from 2019 related to COVID-19 which continued till 2022 [116]. Wang and Huang [117] conducted a bibliometrics analysis of the impact of COVID-19 on sustainability and SDGs. The results revealed COVID-19 pandemic had negative effects on 17 SDGs goals, whereas the pandemic may also bring opportunities to another 14 SDGs goals. D’Adamo et al. [118], analysing 35 indicators related to the economic Sustainable Development Goals for 27 EU countries, observe that northern and western countries outperform other EU member states (Sweden and Denmark gain the highest ranks). Moreover, Resce and Schiltz [119], using means of Hierarchical Stochastic Multicriteria Acceptability Analysis for EU countries, show that Denmark outperforms other EU states, while in Romania and Bulgaria, lower performance levels are observed. Similarly, Ricciolini et al. [120], applying Multiple Reference Point Weak-Strong Composite Indicators to assess UN 2030 Agenda fulfilment, find that Nordic countries reach a good level of global sustainability. Ranjbari *et al*. [121] examined the COVID-19 effect on the triple sustainability pillars (i.e., economic, social, and environmental perspectives) and the SDGs. The study identified the current research gaps and proposed some research directions for sustainable development.

These indices serve as crucial tools for policymakers in devising efficient strategies to enhance a country’s preparedness and prevention measures against potential future pandemics.

The above-presented literature review indicates that plenty of research studies focus on single factors affecting country risk or readiness for the COVID-19 pandemic. In the paper, we combine all these issues and build a more complex and coherent picture that models a country’s stance in the context of huge COVID-19 risks and its preparedness for this unexpected external shock.

## Material and methods

The paper aims to identify groups of the European Union countries characterised by a similar COVID-19 Resilience Index (CRI). The self-calculated CR index reflects the countries’ COVID-19 risk and readiness for a crisis, including a pandemic. Moreover, the study detects the factors that significantly differentiate the distinguished groups. The progressive nature of our methodology to accommodate more factors and the inclusion of a matrix makes it an essential tool for decision-makers when formulating recommendations to reduce the risks of future pandemics and adaptive measures that can be put in place. Additionally, it provides a baseline for attaching compensation funds in future social aid for affected countries from the EU social fund.

Selected indicators are based on data quantified for the CRI indicators for all the EU countries, apart from Cyprus (due to data availability). Data for the CRI are characterised by transparency, reliability, and consistency criteria. CRI ’s framework structure breaks into the measure of COVID-19 risk and the country’s readiness. The COVID-19 risk factors, as presented in the literature review, are divided into three main sub-criteria, i.e., hazards, vulnerability, and exposure, as summarised in **Table 1**.

**Table 1:** COVID risk indicators.

Criteria	Applicable Indicators
**Hazard**	Infected population percentages
	Non-immunised population percentages
	Residential human mobility changes
**Vulnerability**	Population density
	Daily consumption
	Urban concentration
	The stringency of the anti-COVID-19 policy
	Pollution ratio
	Material Footprint
**Exposure**	Elderly people (over 65)
	Diabetes

Source: own elaboration based on the literature review.

As a primary measure of COVID-19 hazard, we use the percentage of the infected population to the total population for each analysed country. The overall population immunity is determined as the sum of protection levels in vaccinated persons and those previously infected but not vaccinated. In the study, we apply data concerning the non-immunised population percentage. We assume that the non-immunised population positively relates to the risk factor as a high value indicates considerable risk. Data come from Refinitiv DataStream and cover 2020.

Additionally, as a measure of COVID-19 hazard, we apply human mobility changes data from COVID-19-related Community Mobility Reports produced by Google [122]. We assume that increased positive changes in human mobility will decrease the risk of COVID-19. We estimate the ratio of changes in human mobility by averaging the daily changes in human mobility. We select the biweekly periods characterised by the highest severity of anti-COVID-19 policy, as we believe that the stringency of the government policy determines human mobility changes during the COVID-19 pandemic [101]. Based on the literature review, we assume that positive human mobility changes in the residential category reduce the COVID-19 hazard ratio. Vulnerability factors refer to countries’ physical situation and risk toward COVID-19 cognisance. We apply population density as one of the vulnerability indicators collected from the Eurostat database for 2020, assuming that a high value leads to considerable risk. Moreover, we used the developed BlavatNik School’s Government Stringency index, which measures the severity of the government’s anti-COVID-19 policy. The index demonstrates the government’s stringency of the responses to the COVID-19 pandemic. The index takes values from 0 to 100 [123]. Higher values express more stringent anti-COVID-19 government policy. Based on the literature review, we assume that the stringency of government restriction and lockdown reduces the COVID-19 vulnerability ratio, and it has a negative relation to the risk as a high value indicated minimal risk. To measure diet habits, we apply the daily consumption of fruit and vegetables from the Eurostat database. We assume it negatively affects the risk as a high daily consumption value indicated minimal COVID-19 risk. The environmental factors are represented by pollution ratio, urban concentration, and material footprint. Data come from the World Bank and Eurostat. We assume that the increased value of these indicators is linked to the higher COVID-19 risk. The remaining risk factors refer to exposure indicators, i.e., ratios of elderly people and diabetes in the total population. Data come from Eurostat and Refinitiv DataStream.

Readiness factors comprehend three components: economic readiness, governance readiness, and social readiness. The applied indicators are presented in **Table 2**. All these indicators are collected from the Sustainable Governance Indicators (SGI) report, provided by the OECD and EU research entities, as one source to support the reliability and validity of the results. However, this does not imply that the CRI is a subset of sustainability, as other dimensions are not factored. The study seeks to establish a resilience index that decision-makers can use as an early warning for a threat to their sustainability path.

**Table 2:** Readiness indicators.

Criteria	Applicable Indicators	
**Economic**	GDP per Capita	
	Labour Markets	
	Primary Balance	
**Governance**	Quality of Democracy	Rule of Law
		Access to Information
	Executive Capacity	Implementation
		Adaptability
**Social**	Gini Coefficient	
	Tertiary Attainment	
	Research, Innovation, and Infrastructure	
	Spending on Preventive Health Programs	

Source: own elaboration based on the literature review.

As depicted in **Table 2**, economic readiness relates to investment. It facilitates the mobilisation of capital from the public and private sectors that could be measured through the ability to do business. The applied indicators are GDP per capita, labour policy, and primary balance. The SGI report’s economic policy indicators aim to stimulate competition and strengthen market principles. According to the theoretical discussion presented earlier in this paper and the concept of the report, these policies offer the greatest benefit to the greatest number of people if they are accompanied by redistributive tax and labour-market redistribution policies and supported by social policies that facilitate equitable societal distribution of the benefits of economic growth. Therefore, they all positively relate to the readiness level, as high values indicate high readiness.

The governance readiness measures the social stability and institutional arrangements contributing to the investment risks. A stable country with a high governance capacity reassures investors that the invested capital could grow with the help of responsive public services and without significant interruption. The following indicators measure it into two subgroups: Quality of Democracy, encompassing the Rule of Law (based on Legal Certainty, Judicial Review, Appointment of Justices, and Corruption Prevention), and Access to Information (measured by Media Freedom, Media Pluralism, Access to Government Information); Executive Capacity, including Implementation (indicated through Government Effectiveness, Ministerial Compliance, Monitoring Ministries, Monitoring Agencies/Bureaucracies, Task Funding, Constitutional Discretion, National Standards, Effective Regulatory Enforcement), and Adaptability (through Domestic Adaptability, International Coordination). All these indicators have a positive relationship with the level of readiness, as high values indicate a high level of readiness.

Social readiness helps society make efficient and equitable use of investment and yield more benefit from the investment, measured by the following indicators: Gini Coefficient, Tertiary Attainment; Research, Innovation, and Infrastructure; Spending on Preventive Health Programs. In addition, the social readiness indicators aim to improve sustainability, thus guaranteeing the long-term sustainability of social welfare systems. All these indicators positively affect the readiness level, as high values indicate high readiness.

We develop a CRI matrix to cluster the countries according to their COVID-19 risk and readiness factors. It is essential to standardise data when working with multidimensional indicators with different units and dimensions [124]. Standardisation in developing composite indices transforms the indicator into a uniform scale and numbers without units that facilitate comparison [125]. The min-max normalisation method (rescaling method), as outlined by Mazziotta and Pareto [126], is applied to align indicators with both positive and negative relationships to the index, thus reducing the extremism effect. Resizing is chosen for its ease of application and ability to eliminate extreme values, thus eliminating partially *Xi* standardised.

The min-max conversion method resizes the different indicators (Xi) in the same range (0–1) based on the minimum (*X_min_*) and maximum (*X_max_*) as presented in Eq 1.

$$"\mathrm{score}"=\left|"\mathrm{direction}"-\frac{\;data-reference\;point}{baseline\;maximum-baseline\;minimum}\right|$$ (1)

The "direction" parameter has two values of 0 or 1. 0 applies when the indicator has an inverse correlation to the final readiness or risk scale. In contrast, 1, when the indicator has a positive correlation to the final readiness or risk scale, a higher risk score means a higher level of a country’s risk and a higher readiness score indicates a higher level of a country’s readiness. CRI is computed by subtracting the risk score from the readiness score for each country and scaling it to a value of 0–100. Eq 2 depicts the formula applied to estimate the CRI index.

CRI=(readinessscore−riskscore+1)*50 (2)

**Fig 1:**
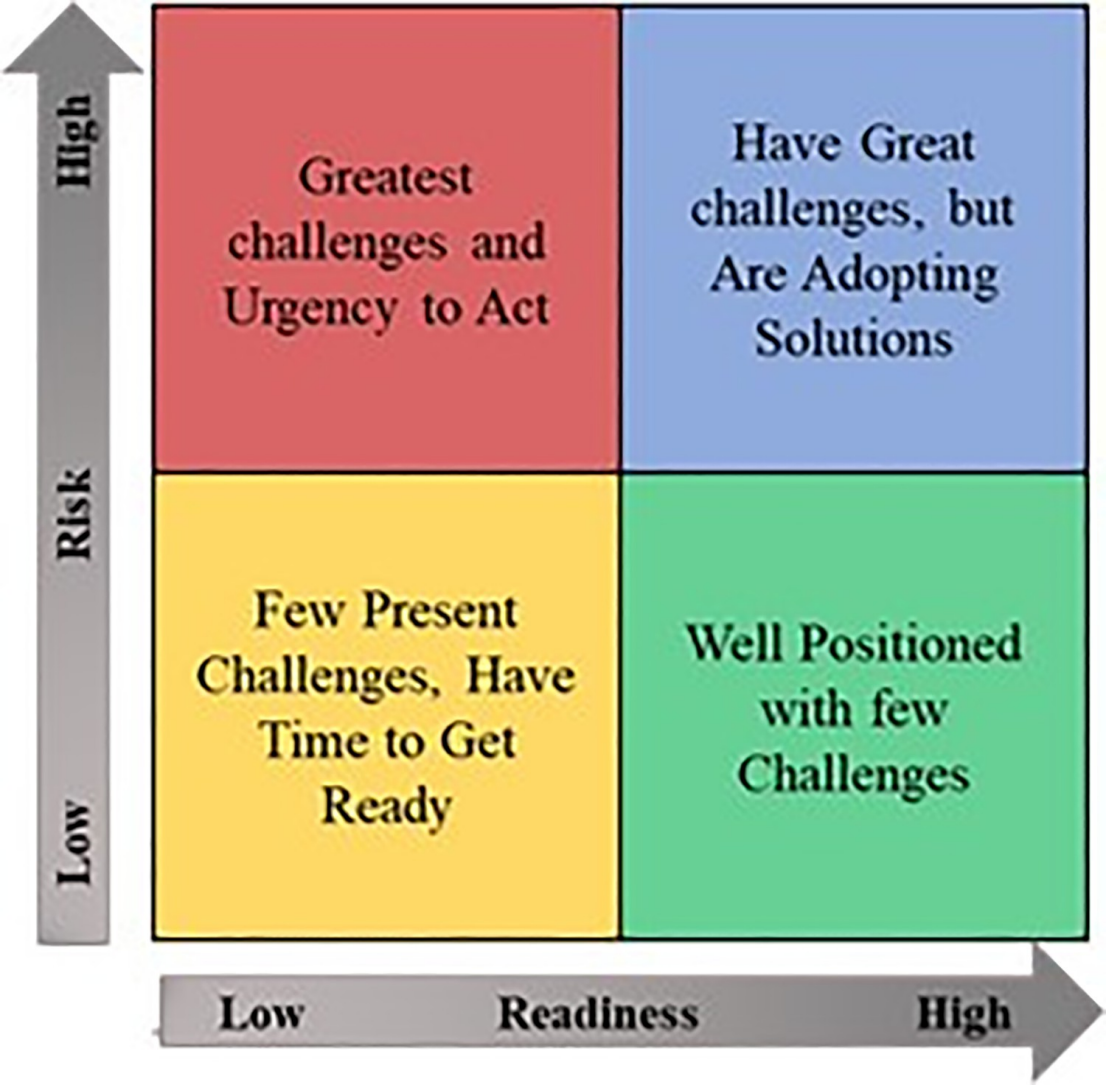
CRI matrix. Source: Authors’ edition based on Cheng *et al*. [127].

The CRI index represents a scatter plot of readiness versus risk. The Matrix provides a visual tool for rapidly comparing countries and monitoring their progress over time. The plot is divided into four quadrants, delineated by the median risk score across all the countries, and the median readiness score is calculated the same way. About half of the countries are to the left of the readiness median and half to the right. Likewise, half fall above the risk median and half below. (Fig 1) presents the CRI matrix. The interpretation of the quadrants is as follows:

- Red (Upper Left) Quadrant (countries facing the most significant challenges and the urgency to act): countries with a high level of COVID-19 risk but a low level of readiness. They are all in dire need of investment to improve their readiness.
- Blue (Upper Right) Quadrant (countries facing significant challenges but are adopting solutions): countries with a high level of COVID-19 risk and a high level of readiness. Adapting is essential in these countries, but they are ready to respond. Perhaps the private sector is more likely to be involved in adaptation here than in low-readiness countries.
- Yellow (Lower Left) Quadrant (countries facing few present challenges, have time to prepare): countries with low readiness levels and COVID-19 risk. Although their risk is relatively low, their adaptation may be delayed due to lower readiness.
- Green (Lower Right) Quadrant (countries well-positioned with few challenges): countries with a low level of COVID-19 risk and a high level of readiness. These countries still need to adapt (none have a perfect COVID-19 risk score), but they are well-prepared.

We assess the existence of significant differences among the distinct groups by applying the Kruskal-Wallis test [128, 129] and the Wilcoxon rank-sum pairwise comparison test [130] with the p-values adjustment using the Benjamini and Hochberg method [131]. The p-values adjustment in the pairwise comparison test was estimated in R software. In the Kruskal-Wallis test, distinguished country groups represent the independent qualitative variable. In contrast, the dependent variable is the selected indicator of countries’ COVID-19 risk factors or selected measures of countries’ readiness for a crisis.

## Results and discussion

The CRI matrix scales using the proximity-to-reference point approach, which scores the level of risk and readiness by the distance to the ideal status (i.e., least risk is 0 and most ready is 1). 0 for risk or 1 for readiness is considered a "full score". Measurement scores can be used to evaluate the distance from the desired condition. The reference points in CRI for individual measures are presented in **Table 3**.

**Table 3:** Reference points for individual indicators.

Sector		Indicator		Reference point	Baseline Min	Baseline Max	Q1	Median (Q2)	Q3	IQR
**COVID-19 risk**	**Hazard**	Infected population percentages (%)		3.61	0.56	6.72	1.98	3.04	3.60	1.62
		Non-vaccinated population percentages (%)		91.03	75.69	99.69	88.25	89.83	90.97	2.72
		Residential human mobility changes (%)		14.92	12.92	33.83	17.33	19.67	26.77	9.44
	**Vulnerability**	Stringency of anti-COVID-19 policy (points)		82.00	67.00	96.00	78.50	81.50	86.50	8.00
		Daily consumption of fruit and vegetables [1–4 portions]		58.00	22.30	68.00	44.53	52.65	57.8	13.28
		Pollution ratio (2017) (ppm)		12.00	6.00	21.00	10.00	12.50	16.00	6.00
		Material Footprint (2020) (tonnes per capita)		4.03	1.00	7.61	4.93	5.67	6.60	1.67
		Population Density (Persons/Km^2^)		108.00	18.00	1642	72.25	107.5	144.25	72.00
		Urban Concentration (2020) (%)		59.00	54.00	98.00	64.5	73.00	84.75	20.25
	**Exposure**	Diabetes (% of total population)		6.60	4.20	9.60	5.225	5.75	6.925	1.70
		Elderly people +65 (% of total population)		19.00	14.00	23.00	19.25	20.00	21.00	1.75
**Readiness**	**Economic Policies**	GDP per Capita (US$)		7.55	3.68	10.00	4.82	5.72	7.14	2.32
		Labour Markets (ratio)		7.37	3.23	8.62	5.66	6.73	7.58	1.93
		Primary Balance (US$)		6.27	3.97	7.68	5.46	5.76	6.61	1.14
	**Social Policies**	Research, Innovation, and Infrastructure (ratio)		6.18	2.75	8.97	4.24	5.06	6.41	2.17
		Tertiary Attainment (%)		6.01	3.73	8.01	4.96	5.97	7.02	2.06
		Gini Coefficient (ratio)		6.24	1.91	8.38	4.47	5.85	6.24	1.76
		Spending on Preventive Health Programs (US$)		4.62	2.59	8.20	4.50	5.24	6.17	1.67
	**Governance Policies**	Quality of Democracy	Access to information (ratio)	6.67	3.00	10.00	5.83	7.00	8.17	2.33
			Rule of Law (ratio)	8.25	3.00	9.75	5.81	7.13	8.00	2.19
		Executive Capacity	Implementation (ratio)	6.83	3.91	8.41	5.10	6.46	7.08	1.98
			Adaptability (ratio)	5.50	3.50	9.50	5.13	6.25	7.88	2.75

Source: own calculation.

**Fig 2:**
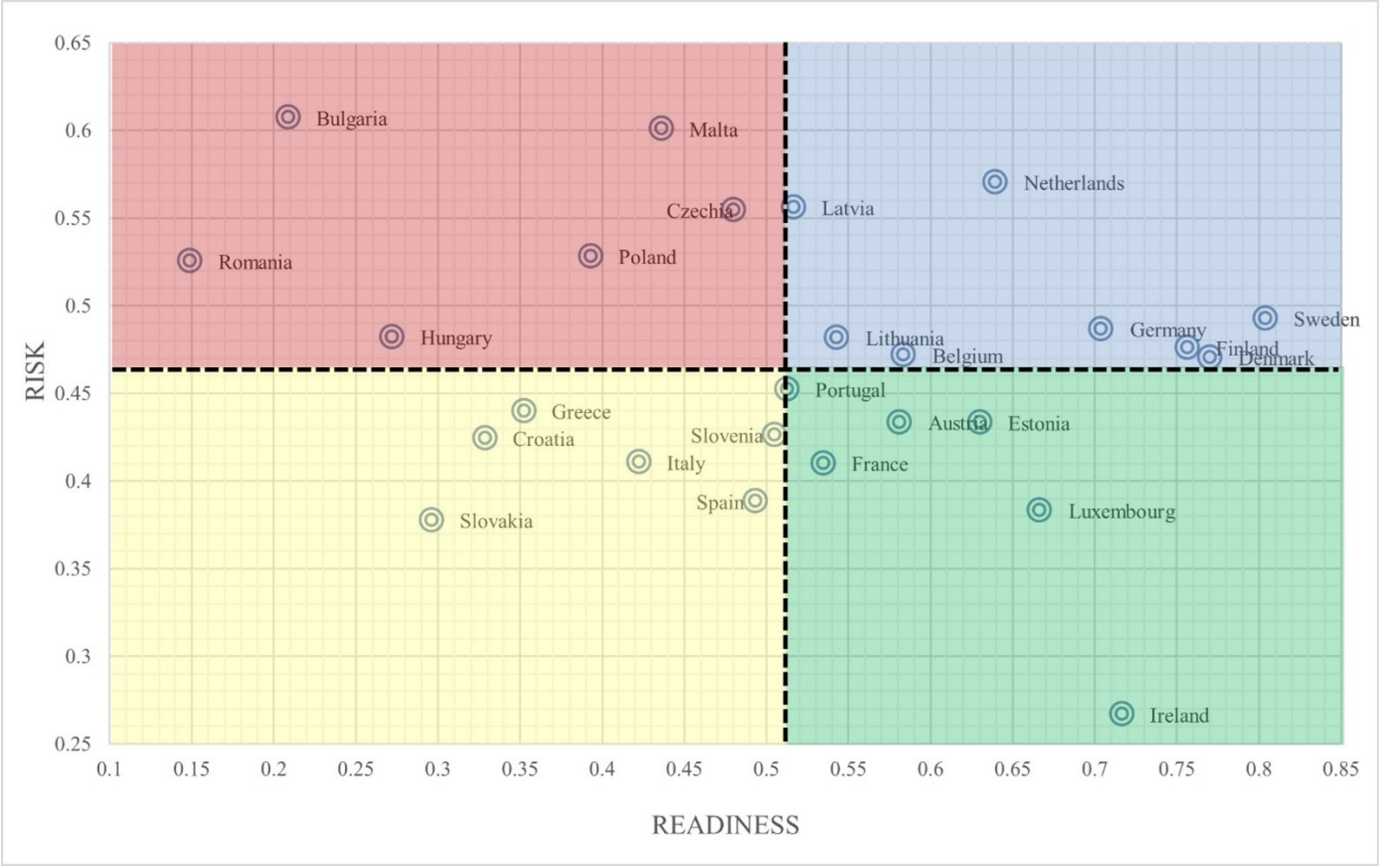
European countries’ distribution in the CRI matrix. Source: Authors’ edition.

Fig 2 depicts the distribution of countries in the CRI matrix. Group I (upper left) refers to countries with high COVID-19 risk and low readiness for a crisis situation (risk of more than 0.467 and readiness of less than 0.512). Group I comprehends such countries as Bulgaria, Romania, Hungary, Poland, Czechia Republic, and Malta. These countries are the greatest challenges, and the urgency to act with significantly high individual indicator values of risk, such as pollution and material footprint, and low value of readiness indicators, such as GDP per capita, Gini coefficient, and the rule of Law. Group II (Upper Right) refers to countries with high COVID-19 risk and high readiness for a crisis situation (risk with more than 0.467 and more than 0.512). Among the group are Sweden, Netherlands, Lithuania, Latvia, Belgium, Denmark, and Germany. These countries have great challenges but are adopting solutions quite protected or prepared to risk high readiness. Group III (Lower Left) refers to countries characterised by low COVID-19 risk and low readiness for a crisis situation (risk with less than 0.467 and readiness of less than 0.512). The group consists of Italy, Spain, Slovenia, Croatia, Slovakia, and Greece. These countries have few present challenges and have time to prepare to face them relative to their readiness level. Group IV (Lower Right) refers to countries characterised by low COVID-19 risk and high readiness for a crisis situation (a risk with less than 0.467 and readiness of more than 0.512). The group includes countries such as Luxembourg, France, Austria, Finland, Estonia, Portugal, and Ireland. As **Table 4** shows the COVID-19 resilience index score for the European countries.

**Table 4:** European COVID-19 resilience index score.

Countries	Readiness Score	Risk Score	CRI Score	Countries	Readiness Score	Risk Score	CRI Score
**Austria**	0.58	0.43	57.38	**Italy**	0.42	0.41	50.61
**Belgium**	0.58	0.47	55.60	**Latvia**	0.52	0.56	48.04
**Bulgaria**	0.21	0.61	30.09	**Lithuania**	0.54	0.48	53.07
**Croatia**	0.33	0.42	45.23	**Luxembourg**	0.67	0.38	64.17
**Czechia**	0.48	0.55	46.29	**Malta**	0.44	0.60	41.78
**Denmark**	0.77	0.47	64.99	**Netherlands**	0.64	0.57	53.45
**Estonia**	0.63	0.43	59.85	**Poland**	0.39	0.53	43.26
**Finland**	0.76	0.48	64.04	**Portugal**	0.51	0.45	53.02
**France**	0.54	0.41	56.25	**Romania**	0.15	0.53	31.19
**Germany**	0.70	0.49	60.87	**Slovakia**	0.29	0.38	45.94
**Greece**	0.35	0.44	45.63	**Slovenia**	0.51	0.43	53.95
**Hungary**	0.27	0.48	39.50	**Spain**	0.49	0.39	55.25
**Ireland**	0.72	0.27	72.51	**Sweden**	0.80	0.49	65.58

Source: own calculation.

We apply the Kruskal-Wallis test to assess significant differences between obtained groups. We use the test to check which factors related to the COVID-19 risk and the country’s readiness for a crisis statistically significantly differentiate the selected country groups. The results of this analysis will help us identify factors that seem to be the most important in terms of COVID-19 risk reduction and countries’ readiness for the crisis increase.

The above-presented country groups represent the independent qualitative variable in the Kruskal-Wallis test. In contrast, the dependent variable is the selected indicator of countries’ COVID-19 risk factors and selected measures of countries’ readiness for a crisis. Table 5 presents the results of the Kruskal-Wallis test for COVID-19 risk indicators.

**Table 5:** The Kruskal-Wallis test results for COVID-19 risk indicators.

COVID-19 risk indicators	K-W Chi-squared test statistic	p-value
**Infected population percentages**	1.04	0.791
**Non-vaccinated population percentages**	2.30	0.513
**Residential human mobility changes**	8.85	0.031
**The stringency of the anti-COVID-19 policy**	10.28	0.016
**Daily consumption of fruit and vegetables**	7.05	0.071
**Pollution ratio**	12.77	0.005
**Material Footprint**	9.14	0.027
**Population Density**	0.45	0.929
**Urban concentration**	5.85	0.119
**Diabetes**	2.77	0.429
**Elderly people +65 (% of the total population)**	6.08	0.108

Source: own calculation.

The research results in **Table 5** indicate four COVID-19 risk indicators that significantly, at a 0.05 significance level, differentiate the countries in the first year of the COVID-19 pandemic. Significant factors are the changes in residential human mobility, the stringency of anti-COVID-19 policy, pollution, and material footprint. Additionally, we use a pairwise comparison Wilcoxon rank-sum test to check whether the detected significant differences refer to all four country groups or selected ones. The results of the pairwise comparison test are presented in **Table 6**.

**Table 6:** Pairwise comparison of Wilcoxon rank-sum test results for COVID-19 risk indicators.

Pairs	Pairwise comparison (p-value)–Wilcoxon rank-sum test			
	Residential human mobility changes	The stringency of the anti-COVID-19 policy	Pollution	Material Footprint
**I-II**	0.262	0.178	0.013	0.042
**I-III**	0.154	0.178	0.717	0.281
**I-IV**	0.644	0.463	0.023	0.044
**II-III**	0.045	0.026	0.107	0.241
**II-IV**	0.184	0.178	0.654	0.996
**III-IV**	0.995	0.347	0.107	0.281

Source: own calculation.

**Fig 3:**
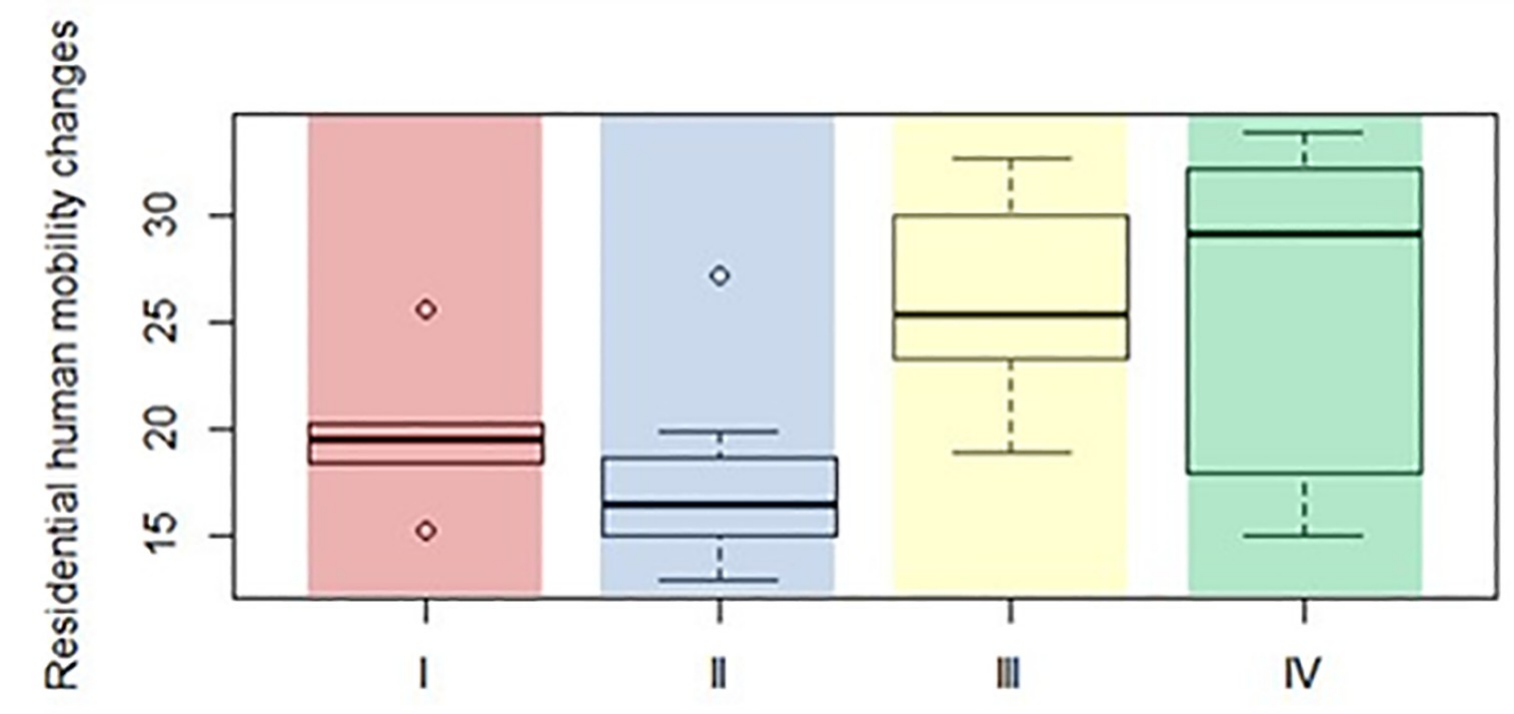
Boxplot for human mobility changes in residential category in four distinguished groups. Source: own calculation based on the data available in the EU.

Table 6 results imply significant differences, at a 5% significance level, in the median level of human mobility changes in the residential category between country groups II and III, i.e., countries with a high risk of COVID-19 and high level of readiness and low risk of COVID-19 and low level of readiness. Moreover, (Fig 3) shows that countries with high COVID-19 risk (groups I and II) are characterised by, on average, smaller residential mobility increases than countries with low COVID-19 risk groups (groups III and IV). It indicates that greater changes in human mobility and greater people’s flexibility in adapting to remote work may lower the risk of rapid COVID-19 disease spread.

**Fig 4:**
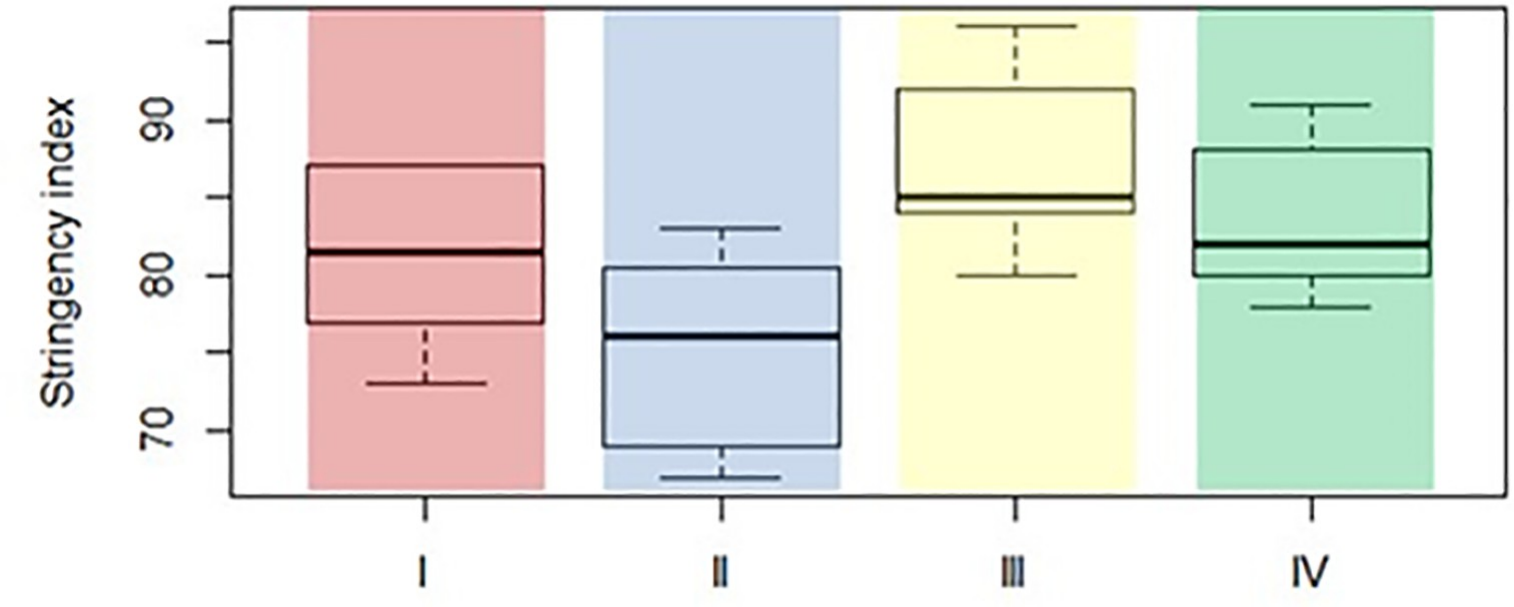
Boxplot for stringency index category in four distinguished groups. Source: own calculation based on the data available in the EU.

The pairwise comparison Wilcoxon rank-sum test results reveal the significant differences, at a 5% significance level, in the median level of the stringency level of anti-COVID-19 policy between country groups II and III. Additionally, (Fig 4) shows that countries with high COVID-19 risk (groups I and II) are characterised by a lower average level of government anti-COVID-19 restrictions stringency than countries with low COVID-19 risk groups (groups III and IV). It implies that a more stringent anti-COVID-19 policy could reduce the risk associated with COVID-19.

The results show that low-COVID-19-risk country groups are characterised by greater positive residential mobility changes and more stringent anti-COVID-19 government policies. It corresponds to [101], who reveals a positive relationship between the stringency of anti-COVID-19 government policy and human mobility changes in the residential category.

The pairwise comparison Wilcoxon rank-sum test results reveal the significant differences, at a 5% significance level, in the median level of pollution and material footprint factors between groups I and II, and groups I and IV. Moreover, it shows that these two factors do not only differentiate the countries in terms of COVID-19 risk but also in terms of their readiness for a crisis. For the rest of the boxplots, see **S1 Appendix**.

The research results in **Table 7** indicate eight out of eleven country readiness factors that significantly differentiate the analysed country groups at a 5% significance level. Among the significant factors are the economic indicators, such as GDP per capita and Labour markets, the governance indicators, such as Rule of Law, Access to Information, Implementation and Adaptability measures; and social indicators, such as Tertiary Attainment and Research, innovation, and infrastructure. We apply a pairwise comparison Wilcoxon rank-sum test to check whether the detected significant differences refer to all four country groups or selected ones. The results of the pairwise comparison test are presented in **Table 8**.

**Table 7:** Kruskal-Wallis test results for countries’ readiness factors related to economic, governance and social indicators.

	K-W Chi-squared test statistic	p-value
**GDP per Capita**	11.69	0.008
**Labour markets**	10.89	0.012
**Primary balance**	5.76	0.124
**Rule of Law**	16.25	0.001
**Access to Information**	14.13	0.003
**Implementation**	15.93	0.001
**Adaptability**	13.49	0.004
**Gini Coefficient**	0.35	0.951
**Tertiary Attainment**	16.15	0.001
**Research, Innovation, and Infrastructure (RII)**	16.20	0.001
**Spending on Preventive Health Programs**	2.16	0.540

Source: own calculation.

**Table 8:** Pairwise comparison of Wilcoxon rank-sum test results for COVID-19 risk indicators.

Pairs	Pairwise comparison (p-value)–Wilcoxon rank-sum test							
	GDP per Capita	Labour markets	Rule of Law	Access to Information	Implementation	Adaptability	Tertiary Attainment	R&I and Infrastructure
**I-II**	0.036	0.172	0.022	0.018	0.012	0.014	0.016	0.012
**I-III**	0.829	0.172	0.046	0.078	0.999	0.547	0.616	0.773
**I-IV**	0.036	0.172	0.027	0.070	0.012	0.044	0.016	0.012
**II-III**	0.036	0.044	0.027	0.018	0.012	0.045	0.016	0.012
**II-IV**	0.555	0.941	0.160	0.252	0.496	0.604	0.616	0.409
**III-IV**	0.036	0.044	0.104	0.320	0.012	0.116	0.022	0.012

Source: own calculation.

The results of the pairwise comparison Wilcoxon rank-sum test reveal the significant differences, at a 5% significance level, in the median level of eight country readiness factors mainly between the high- and low- readiness group of countries, i.e., between I and II, I and IV, II and III, and III and IV. Moreover, the boxplots (**S1 Appendix**) show that high readiness countries are characterised by the highest values of indicators such as level of GDP per capita, Labour markets, Rule of Law, Access to Information, Implementation and Adaptability measures, Tertiary Attainment and Research, Innovation, and Infrastructure ratio.

The Kruskal-Wallis test research results might be applied to draw some recommendations for decision-makers. However, it should be emphasised that the research results might be affected by the time period of the study. Data for risk factors cover only the first year of the COVID-19 pandemic, i.e., 2020, while the level of country readiness was presented based on data from the period before the COVID-19 pandemic.

## Conclusions

Handling complex issues in a diverse environment requires simple approaches that can be synthesised quickly and effectively. Furthermore, approaches that transcend beyond the scope of any sector, as the pandemic and future crisis prove to be borderless. Therefore, a multi-sectoral and stakeholder approach that can ensure the European Union achieves a just transition is fundamental in the face of any challenge. Moreover, designing such approaches is a subset of a multi-perspective approach that can be compared from a relative development position. Unfortunately, there is no standard approach to compare or handle emergencies. Therefore, improving what already exists forms a strong standpoint for creating a springboard for the EU to handle crises.

COVID-19 has rapidly billowed into a pandemic and swept countries worldwide. Pandemics are considered very low-probability catastrophic events linked to deep uncertainty about their timing and severity. Thus, national decision-makers have limited options for implementing proactive measures for pandemics. The critical problem in the COVID-19 crisis is the measurement of the country’s readiness to cope with the pandemic threats, i.e., to assess: the performance in reducing COVID-19-related mortality, support vaccination programmes to constrain future pandemic threats and support the recovery of socio-economic systems.

The paper aims to identify groups of the European Union countries characterized by a similar COVID-19 Resilience Index (CRI). The CRI index reflects the countries’ COVID-19 risk and their readiness for a crisis, including future pandemics. Additionally, the study identifies factors that differentiate the distinguished groups where Bulgaria, Hungary, Malta, and Poland have the lowest COVID-19 Resilience Index score while Luxembourg, Sweden and Denmark have the Highest CRI.

We believe that the COVID-19 Resilience Index in the European Union countries might constitute an effective tool to distinguish the countries according to the risk and readiness levels related to a crisis. The calculated CRI is meant to help the decision-makers either improve the country’s status or tackle their risk by implementing the right policies targeting sustainable development under different circumstances and crises.

The Kruskal-Wallis test results indicate four COVID-19 risk indicators that significantly differentiate the countries in the first year of the COVID-19 pandemic. As it might distinguish the level of restrictions and the policies level, the study has shown a significant correlation to the environmental factors. Therefore, the levels of intervention and decision-making vary accordingly. Many precautions can be taken at the country and individual levels. Thus, more environmental policies are needed to enrich the quality of life and reduce life risks. Besides the environmental factors, the study emphasised the importance of the Rule of Law, which affected the disease spread and illustrated the power to control the risk. We might state that generally, European countries’ advantage is the restrictive rules, and the real risk is concentrated around environmental issues such as climatic and ecological.

Some recommendations to enlighten the decision-makers based on the study would be as follows, countries such as Bulgaria, Romania, Hungary, Poland, Czechia Republic, and Malta are in need to work on improving their indicators such as GDP per capita, Gini coefficient, and the rule of Law. Whilst these countries (Sweden, Netherlands, Lithuania, Latvia, Belgium, Denmark, and Germany) have to care about the ecological standers to tackle the COVID risks. However, the group consisting of Italy, Spain, Slovenia, Croatia, Slovakia, and Greece are doing fine just now, their advantage is all about time, and they should keep the balance up. The Country Resilience Index (CRI) application for another crisis or research group is challenging for future research. However, based on our findings and the classification by CRI for the EU, we can conclude that there is a higher likelihood of a positive correlation between our CRI ranking and progress on SDG. Thus, there is a need to research the relationship in the future and expound on how the CRI could be used to inform decision-making for sustainable development or even how sustainable development implementation could be implemented to improve countries’ resilience to hazards and disasters. However, our CRI indicators are just a tiny portion of defining sustainability as they could vary from system to system.

## Supporting information

S1 Appendix (DOCX)

S1 Data (XLSX)

## References

[1] Zhang Q., Gao J., Wu J. T., Cao Z., and Dajun Zeng D., ‘Data science approaches to confronting the COVID-19 pandemic: a narrative review’, *Philos*. *Trans*. *R*. *Soc*. *Math*. *Phys*. *Eng*. *Sci*., vol. 380, no. 2214, p. 20210127, doi: 10.1098/rsta.2021.0127 Jan. 2022. PMC8607150 34802267

[2] Leach M., MacGregor H., Scoones I., and Wilkinson A., ‘Post-pandemic transformations: How and why COVID-19 requires us to rethink development’, *World Dev*., vol. 138, p. 105233, Feb. 2021, doi: 10.1016/j.worlddev.2020.105233 33100478 PMC7566764

[3] Qazi A. and Simsekler M. C. E., ‘Nexus between drivers of COVID-19 and country risks’, *Socioecon*. *Plann*. *Sci*., p. 101276, Feb. 2022, doi: 10.1016/j.seps.2022.101276 35228762 PMC8864897

[4] Nakat Z. and Bou-Mitri C., ‘COVID-19 and the food industry: Readiness assessment’, *Food Control*, vol. 121, p. 107661, Mar. 2021, doi: 10.1016/j.foodcont.2020.107661 33013004 PMC7523550

[5] Dembech M., Katz Z., and Szilard I., ‘Strengthening Country Readiness for Pandemic-Related Mass Movement: Policy Lessons Learned’, *Int*. *J*. *Environ*. *Res*. *Public*. *Health*, vol. 18, no. 12, p. 6377, Jun. 2021, doi: 10.3390/ijerph18126377 34204689 PMC8296205

[6] WHO, ‘WHO Director-General’s opening remarks at the media briefing on COVID-19–23 October 2020’, 2020. https://www.who.int/director-general/speeches/detail/who-director-general-s-opening-remarks-at-the-media-briefing-on-covid-19—23-october-2020 (accessed Jun. 27, 2022).

[7] Qazi A., Simsekler M. C. E., and Gaudenzi B., ‘Prioritizing Multidimensional Interdependent Factors Influencing COVID‐19 Risk’, *Risk Anal*., vol. 42, no. 1, pp. 143–161, Jan. 2022, doi: 10.1111/risa.13841 34664727 PMC8661737

[8] Qazi A., Simsekler M. C. E., and Akram M., ‘Efficacy of early warning systems in assessing country-level risk exposure to COVID-19’, *Geomat*. *Nat*. *Hazards Risk*, vol. 12, no. 1, pp. 2352–2366, Jan. 2021, doi: 10.1080/19475705.2021.1962984

[9] Haghani M., Bliemer M. C. J., Goerlandt F., and Li J., ‘The scientific literature on Coronaviruses, COVID-19 and its associated safety-related research dimensions: A scientometric analysis and scoping review’, *Saf*. *Sci*., vol. 129, p. 104806, Sep. 2020, doi: 10.1016/j.ssci.2020.104806 32382213 PMC7203062

[10] Coccia M., ‘Factors determining the diffusion of COVID-19 and suggested strategy to prevent future accelerated viral infectivity similar to COVID’, *Sci*. *Total Environ*., vol. 729, p. 138474, Aug. 2020, doi: 10.1016/j.scitotenv.2020.138474 32498152 PMC7169901

[11] Coccia M., ‘Preparedness of countries to face COVID-19 pandemic crisis: Strategic positioning and factors supporting effective strategies of prevention of pandemic threats’, *Environ*. *Res*., vol. 203, p. 111678, Jan. 2022, doi: 10.1016/j.envres.2021.111678 34280421 PMC8284056

[12] Sirleaf E. J. and Clark H., ‘Report of the Independent Panel for Pandemic Preparedness and Response: making COVID-19 the last pandemic’, *The Lancet*, vol. 398, no. 10295, pp. 101–103, Jul. 2021, doi: 10.1016/S0140-6736(21)01095-3 33991477 PMC9751704

[13] Brooks N., Neil Adger W., and Mick Kelly P., ‘The determinants of vulnerability and adaptive capacity at the national level and the implications for adaptation’, *Glob*. *Environ*. *Change*, vol. 15, no. 2, pp. 151–163, Jul. 2005, doi: 10.1016/j.gloenvcha.2004.12.006

[14] Peduzzi P., Dao H., Herold C., and Mouton F., ‘Assessing global exposure and vulnerability towards natural hazards: the Disaster Risk Index’, *Nat*. *Hazards Earth Syst*. *Sci*., vol. 9, no. 4, pp. 1149–1159, Jul. 2009, doi: 10.5194/nhess-9-1149-2009

[15] Cardona O.-D. et al., ‘Determinants of Risk: Exposure and Vulnerability’, in *Managing the Risks of Extreme Events and Disasters to Advance Climate Change Adaptation*, Field C. B., Barros V, Stocker T. F., and Dahe Q, Eds., 1st ed.Cambridge University Press, 2012, pp. 65–108. Accessed: Jun. 27, 2022. [Online]. Available: https://www.cambridge.org/core/product/identifier/CBO9781139177245A021/type/book_part

[16] Seddighi H., ‘COVID-19 as a Natural Disaster: Focusing on Exposure and Vulnerability for Response’, *Disaster Med*. *Public Health Prep*., vol. 14, no. 4, pp. e42–e43, Aug. 2020, doi: 10.1017/dmp.2020.279 32713408 PMC7492580

[17] de la Fuente-Mella H., Rubilar R., Chahuán-Jiménez K., and Leiva V., ‘Modeling COVID-19 Cases Statistically and Evaluating Their Effect on the Economy of Countries’, *Mathematics*, vol. 9, no. 13, p. 1558, Jul. 2021, doi: 10.3390/math9131558

[18] Jones D. and Helmreich S., ‘A history of herd immunity’, *The Lancet*, vol. 396, no. 10254, pp. 810–811, Sep. 2020, doi: 10.1016/S0140-6736(20)31924-3 32950081 PMC7498207

[19] Aschwanden C., ‘Five reasons why COVID herd immunity is probably impossible’, *Nature*, vol. 591, no. 7851, pp. 520–522, Mar. 2021, doi: 10.1038/d41586-021-00728-2 33737753

[20] Yadegari I., Omidi M., and Smith S. R., ‘The herd-immunity threshold must be updated for multi-vaccine strategies and multiple variants’, *Sci*. *Rep*., vol. 11, no. 1, p. 22970, Dec. 2021, doi: 10.1038/s41598-021-00083-2 34836984 PMC8626504

[21] García-García D., Morales E., Fonfría E. S., Vigo I., and Bordehore C., ‘Caveats on COVID-19 herd immunity threshold: the Spain case’, *Sci*. *Rep*., vol. 12, no. 1, p. 598, Dec. 2022, doi: 10.1038/s41598-021-04440-z 35022463 PMC8755751

[22] Sigler T. et al., ‘The socio-spatial determinants of COVID-19 diffusion: the impact of globalisation, settlement characteristics and population’, *Glob*. *Health*, vol. 17, no. 1, p. 56, Dec. 2021, doi: 10.1186/s12992-021-00707-2 34016145 PMC8135172

[23] Chinazzi M. et al., ‘The effect of travel restrictions on the spread of the 2019 novel coronavirus (COVID-19) outbreak’, *Science*, vol. 368, no. 6489, pp. 395–400, Apr. 2020, doi: 10.1126/science.aba9757 32144116 PMC7164386

[24] Zhu D., Mishra S. R., Han X., and Santo K., ‘Social distancing in Latin America during the COVID-19 pandemic: an analysis using the Stringency Index and Google Community Mobility Reports’, *J*. *Travel Med*., vol. 27, no. 8, p. taaa125, Dec. 2020, doi: 10.1093/jtm/taaa125 32729931 PMC7454760

[25] Saha J., Barman B., and Chouhan P., ‘Lockdown for COVID-19 and its impact on community mobility in India: An analysis of the COVID-19 Community Mobility Reports, 2020’, *Child*. *Youth Serv*. *Rev*., vol. 116, p. 105160, Sep. 2020, doi: 10.1016/j.childyouth.2020.105160 32834269 PMC7289746

[26] Wielechowski M., Czech K., and Grzęda Ł., ‘Decline in Mobility: Public Transport in Poland in the time of the COVID-19 Pandemic’, *Economies*, vol. 8, no. 4, p. 78, Sep. 2020, doi: 10.3390/economies8040078

[27] Teller J., ‘Urban density and Covid-19: towards an adaptive approach’, *Build*. *Cities*, vol. 2, no. 1, pp. 150–165, Feb. 2021, doi: 10.5334/bc.89

[28] Li Q. et al., ‘Early Transmission Dynamics in Wuhan, China, of Novel Coronavirus–Infected Pneumonia’, *N*. *Engl*. *J*. *Med*., vol. 382, no. 13, pp. 1199–1207, Mar. 2020, doi: 10.1056/NEJMoa2001316 31995857 PMC7121484

[29] Chen K. and Li Z., ‘The spread rate of SARS-CoV-2 is strongly associated with population density’, *J*. *Travel Med*., vol. 27, no. 8, p. taaa186, Dec. 2020, doi: 10.1093/jtm/taaa186 33009808 PMC7665678

[30] Rashed E. A., Kodera S., Gomez-Tames J., and Hirata A., ‘Influence of Absolute Humidity, Temperature and Population Density on COVID-19 Spread and Decay Durations: Multi-Prefecture Study in Japan’, *Int*. *J*. *Environ*. *Res*. *Public*. *Health*, vol. 17, no. 15, p. 5354, Jul. 2020, doi: 10.3390/ijerph17155354 32722294 PMC7432865

[31] Bhadra A., Mukherjee A., and Sarkar K., ‘Impact of population density on Covid-19 infected and mortality rate in India’, *Model*. *Earth Syst*. *Environ*., vol. 7, no. 1, pp. 623–629, Mar. 2021, doi: 10.1007/s40808-020-00984-7 33072850 PMC7553801

[32] Ilardi A., Chieffi S., Iavarone A., and Ilardi C. R., ‘SARS-CoV-2 in Italy: Population Density Correlates with Morbidity and Mortality’, *Jpn*. *J*. *Infect*. *Dis*., vol. 74, no. 1, pp. 61–64, Jan. 2021, doi: 10.7883/yoken.JJID.2020.200 32611978

[33] Carozzi F., ‘Urban Density and Covid-19’, *SSRN Electron*. *J*., 2020, doi: 10.2139/ssrn.3643204

[34] Harb M. et al., ‘Integrating Data-Driven and Participatory Modeling to Simulate Future Urban Growth Scenarios: Findings from Monastir, Tunisia’, *Urban Sci*., vol. 4, no. 1, p. 10, Feb. 2020, doi: 10.3390/urbansci4010010

[35] Ali S. H. and Keil R., ‘Global Cities and the Spread of Infectious Disease: The Case of Severe Acute Respiratory Syndrome (SARS) in Toronto, Canada’, *Urban Stud*., vol. 43, no. 3, pp. 491–509, Mar. 2006, doi: 10.1080/00420980500452458

[36] Matthew R. A. and McDonald B., ‘Cities under Siege: Urban Planning and the Threat of Infectious Disease’, *J*. *Am*. *Plann*. *Assoc*., vol. 72, no. 1, pp. 109–117, Mar. 2006, doi: 10.1080/01944360608976728

[37] Shekhar H. et al., ‘Are leading urban centers predisposed to global risks- An analysis of the global south from COVID-19 perspective’, *Habitat Int*., vol. 121, p. 102517, Mar. 2022, doi: 10.1016/j.habitatint.2022.102517 35125583 PMC8801593

[38] Liang D. et al., ‘Urban Air Pollution May Enhance COVID-19 Case-Fatality and Mortality Rates in the United States’, *The Innovation*, vol. 1, no. 3, p. 100047, Nov. 2020, doi: 10.1016/j.xinn.2020.100047 32984861 PMC7505160

[39] Chauhan R. S. et al., ‘COVID-19 related Attitudes and Risk Perceptions across Urban, Rural, and Suburban Areas in the United States’, *Findings*, Jun. 2021, doi: 10.32866/001c.23714

[40] Souch J. M. and Cossman J. S., ‘A Commentary on Rural‐Urban Disparities in COVID‐19 Testing Rates per 100,000 and Risk Factors’, *J*. *Rural Health*, vol. 37, no. 1, pp. 188–190, Jan. 2021, doi: 10.1111/jrh.12450 32282964 PMC7262182

[41] Jayawardena R. and Misra A., ‘Balanced diet is a major casualty in COVID-19’, *Diabetes Metab*. *Syndr*. *Clin*. *Res*. *Rev*., vol. 14, no. 5, pp. 1085–1086, Sep. 2020, doi: 10.1016/j.dsx.2020.07.001 32652495 PMC7333608

[42] Bousquet J. et al., ‘Is diet partly responsible for differences in COVID-19 death rates between and within countries?’, *Clin*. *Transl*. *Allergy*, vol. 10, no. 1, p. 16, Dec. 2020, doi: 10.1186/s13601-020-00323-0 32499909 PMC7250534

[43] Alvarado R., Ponce P., Criollo A., Córdova K., and Khan M. K., ‘Environmental degradation and real per capita output: New evidence at the global level grouping countries by income levels’, *J*. *Clean*. *Prod*., vol. 189, pp. 13–20, Jul. 2018, doi: 10.1016/j.jclepro.2018.04.064

[44] Zhang L., Godil D. I., Bibi M., Khan M. K., Sarwat S., and Anser M. K., ‘Caring for the environment: How human capital, natural resources, and economic growth interact with environmental degradation in Pakistan? A dynamic ARDL approach’, *Sci*. *Total Environ*., vol. 774, p. 145553, Jun. 2021, doi: 10.1016/j.scitotenv.2021.145553 33611006

[45] Zia S. et al., ‘Striving towards environmental sustainability: how natural resources, human capital, financial development, and economic growth interact with ecological footprint in China’, *Environ*. *Sci*. *Pollut*. *Res*., vol. 28, no. 37, pp. 52499–52513, Oct. 2021, doi: 10.1007/s11356-021-14342-2 34013413

[46] European Environment Agency, *COVID-19*: *lessons for sustainability*? LU: Publications Office, 2022. Accessed: Jun. 16, 2022. [Online]. Available: https://data.europa.eu/doi/10.2800/289185

[47] Asdaq S. M. B., Rabbani S. I., Alamri A. S., Alsanie W. F., Alhomrani M., and Al-Yamani M. J., ‘Influence of environmental factors on the spread of COVID-19 in Saudi Arabia’, *PeerJ*, vol. 10, p. e12732, Jan. 2022, doi: 10.7717/peerj.12732 35036101 PMC8743009

[48] Maheswari S., Pethannan R., and Sabarimurugan S., ‘Air pollution enhances susceptibility to novel coronavirus (COVID-19) infection—an impact study’, *Environ*. *Anal*. *Health Toxicol*., vol. 35, no. 4, pp. e2020020–2020020, Dec. 2020, doi: 10.5620/eaht.2020020 33434420 PMC7829407

[49] Comunian S., Dongo D., Milani C., and Palestini P., ‘Air Pollution and COVID-19: The Role of Particulate Matter in the Spread and Increase of COVID-19’s Morbidity and Mortality’, *Int*. *J*. *Environ*. *Res*. *Public*. *Health*, vol. 17, no. 12, p. 4487, Jun. 2020, doi: 10.3390/ijerph17124487 32580440 PMC7345938

[50] Petroni M. et al., ‘Hazardous air pollutant exposure as a contributing factor to COVID-19 mortality in the United States’, *Environ*. *Res*. *Lett*., vol. 15, no. 9, p. 0940a9, Sep. 2020, doi: 10.1088/1748-9326/abaf86

[51] Coccia M., ‘Factors determining the diffusion of COVID-19 and suggested strategy to prevent future accelerated viral infectivity similar to COVID’, *Sci*. *Total Environ*., vol. 729, p. 138474, Aug. 2020, doi: 10.1016/j.scitotenv.2020.138474 32498152 PMC7169901

[52] De Vos J., ‘The effect of COVID-19 and subsequent social distancing on travel behavior’, *Transp*. *Res*. *Interdiscip*. *Perspect*., vol. 5, p. 100121, May 2020, doi: 10.1016/j.trip.2020.100121 34171016 PMC7180344

[53] Koh D., ‘COVID-19 lockdowns throughout the world’, *Occup*. *Med*., vol. 70, no. 5, pp. 322–322, Jul. 2020, doi: 10.1093/occmed/kqaa073

[54] Chisadza C., Clance M., and Gupta R., ‘Government Effectiveness and the COVID-19 Pandemic’, *Sustainability*, vol. 13, no. 6, p. 3042, Mar. 2021, doi: 10.3390/su13063042

[55] Hale T. et al., ‘Government responses and COVID-19 deaths: Global evidence across multiple pandemic waves’, *PLOS ONE*, vol. 16, no. 7, p. e0253116, Jul. 2021, doi: 10.1371/journal.pone.0253116 34242239 PMC8270409

[56] Kraemer M. U. G. et al., ‘The effect of human mobility and control measures on the COVID-19 epidemic in China’, *Science*, vol. 368, no. 6490, pp. 493–497, May 2020, doi: 10.1126/science.abb4218 32213647 PMC7146642

[57] Piquero A. R. and Kurland J., ‘More stringent measures against COVID-19 are associated with lower cases and deaths in Florida and Miami-Dade’, *Am*. *J*. *Emerg*. *Med*., vol. 53, pp. 262–263, Mar. 2022, doi: 10.1016/j.ajem.2021.04.066 33931277 PMC8065232

[58] McGrail D. J., Dai J., McAndrews K. M., and Kalluri R., ‘Enacting national social distancing policies corresponds with dramatic reduction in COVID19 infection rates’, *PLOS ONE*, vol. 15, no. 7, p. e0236619, Jul. 2020, doi: 10.1371/journal.pone.0236619 32730356 PMC7392246

[59] Yang Q.-C., Chen X., Chang C.-P., Chen D., and Hao Y., ‘What is the relationship between government response and COVID-19 pandemics? Global evidence of 118 countries’, *Struct*. *Change Econ*. *Dyn*., vol. 59, pp. 98–107, Dec. 2021, doi: 10.1016/j.strueco.2021.08.007 35317309 PMC8397493

[60] Jinjarak Y., Ahmed R., Nair-Desai S., Xin W., and Aizenman J., ‘Accounting for Global COVID-19 Diffusion Patterns, January–April 2020’, *Econ*. *Disasters Clim*. *Change*, vol. 4, no. 3, pp. 515–559, Oct. 2020, doi: 10.1007/s41885-020-00071-2 32901228 PMC7471593

[61] Chaudhry R., Dranitsaris G., Mubashir T., Bartoszko J., and Riazi S., ‘A country level analysis measuring the impact of government actions, country preparedness and socioeconomic factors on COVID-19 mortality and related health outcomes’, *EClinicalMedicine*, vol. 25, p. 100464, Aug. 2020, doi: 10.1016/j.eclinm.2020.100464 32838237 PMC7372278

[62] Dhama K. et al., ‘Geriatric Population During the COVID-19 Pandemic: Problems, Considerations, Exigencies, and Beyond’, *Front*. *Public Health*, vol. 8, p. 574198, Sep. 2020, doi: 10.3389/fpubh.2020.574198 33072713 PMC7536316

[63] Rashedi J. et al., ‘Risk Factors for COVID-19’, *Infez*. *Med*., vol. 28, no. 4, pp. 469–474, Dec. 2020. 33257620

[64] Pizano-Escalante M. G. et al., ‘Direct and Indirect Effects of COVID-19 in Frail Elderly: Interventions and Recommendations’, *J*. *Pers*. *Med*., vol. 11, no. 10, p. 999, Oct. 2021, doi: 10.3390/jpm11100999 34683141 PMC8539433

[65] Rod J. E., Oviedo-Trespalacios O., and Cortes-Ramirez J., ‘A brief-review of the risk factors for covid-19 severity’, *Rev*. *Saúde Pública*, vol. 54, p. 60, Jul. 2020, doi: 10.11606/s1518-8787.2020054002481 32491116 PMC7263798

[66] Peric S. and Stulnig T. M., ‘Diabetes and COVID-19: Disease—Management—People’, *Wien*. *Klin*. *Wochenschr*., vol. 132, no. 13–14, pp. 356–361, Jul. 2020, doi: 10.1007/s00508-020-01672-3 32435867 PMC7238399

[67] Abdi A., Jalilian M., Sarbarzeh P. A., and Vlaisavljevic Z., ‘Diabetes and COVID-19: A systematic review on the current evidences’, *Diabetes Res*. *Clin*. *Pract*., vol. 166, p. 108347, Aug. 2020, doi: 10.1016/j.diabres.2020.108347 32711003 PMC7375314

[68] Schraad-Tischler D. and Seelkopf D., ‘Concept and Methodology: sustainable Governance Indicators’, Bertelsmann Foundation, Gutersloh, 2016.

[69] Eissa N., ‘Pandemic Preparedness and Public Health Expenditure’, *Economies*, vol. 8, no. 3, p. 60, Jul. 2020, doi: 10.3390/economies8030060

[70] Bollyky T. J. et al., ‘Pandemic preparedness and COVID-19: an exploratory analysis of infection and fatality rates, and contextual factors associated with preparedness in 177 countries, from Jan 1, 2020, to Sept 30, 2021’, *The Lancet*, vol. 399, no. 10334, pp. 1489–1512, Apr. 2022, doi: 10.1016/S0140-6736(22)00172-6 35120592 PMC8806194

[71] Gavriluță N., Grecu S.-P., and Chiriac H. C., ‘Sustainability and Employability in the Time of COVID-19. Youth, Education and Entrepreneurship in EU Countries’, *Sustainability*, vol. 14, no. 3, p. 1589, Jan. 2022, doi: 10.3390/su14031589

[72] David A. C. and Pienknagura S., ‘On the effectiveness of containment measures in controlling the COVID-19 pandemic: the role of labour market characteristics and governance’, *Appl*. *Econ*. *Lett*., vol. 28, no. 19, pp. 1641–1647, Nov. 2021, doi: 10.1080/13504851.2020.1841082

[73] He L. et al., ‘Contributions and Challenges of Public Health Social Work Practice during the Initial 2020 COVID-19 Outbreak in China’, *Br*. *J*. *Soc*. *Work*, p. bcac077, Apr. 2022, doi: 10.1093/bjsw/bcac077

[74] Afonso A. and Hauptmeier S., ‘Fiscal Behaviour in the European Union: Rules, Fiscal Decentralization and Government Indebtedness’, *SSRN Electron*. *J*., 2009, doi: 10.2139/ssrn.1399284

[75] Aizenman J., Jinjarak Y., Nguyen H. T. K., and Park D., ‘Fiscal space and government-spending and tax-rate cyclicality patterns: A cross-country comparison, 1960–2016’, *J*. *Macroecon*., vol. 60, pp. 229–252, Jun. 2019, doi: 10.1016/j.jmacro.2019.02.006

[76] Schick A., ‘The role of fiscal rules in budgeting’, *OECD J*. *Budg*., vol. 3, no. 3, pp. 7–34, 2003.

[77] Klose J. and Tillmann P., ‘Stock market response to Covid-19, containment measures and stabilization policies—The case of Europe’, *Int*. *Econ*., vol. 173, pp. 29–44, May 2023, doi: 10.1016/j.inteco.2022.11.004

[78] Ardanaz M., Cavallo E., Izquierdo A., and Puig J., ‘Growth-friendly fiscal rules? Safeguarding public investment from budget cuts through fiscal rule design’, *J*. *Int*. *Money Finance*, vol. 111, p. 102319, Mar. 2021, doi: 10.1016/j.jimonfin.2020.102319

[79] Tevdovski D., Jolakoski P., and Stojkoski V., ‘Determinants of budget deficits: Focus on the effects from the COVID-19 crisis’, 2021, doi: 10.48550/ARXIV.2105.14959

[80] Hochrainer-Stigler S., ‘Changes in fiscal risk against natural disasters due to Covid-19’, *Prog*. *Disaster Sci*., vol. 10, p. 100176, Apr. 2021, doi: 10.1016/j.pdisas.2021.100176

[81] Rawdanowicz Ł., Turban S., Haas J., and Millot V., ‘Measuring environmental policy stringency in OECD countries: An update of the OECD composite EPS indicator’, OECD Economics Department Working Papers 1703, 2021. Accessed: Mar. 20, 2022. [Online]. Available: https://www.oecd-ilibrary.org/economics/measuring-environmental-policy-stringency-in-oecd-countries_90ab82e8-en

[82] Kahn M. E., ‘The Death Toll from Natural Disasters: The Role of Income, Geography, and Institutions’, *Rev*. *Econ*. *Stat*., vol. 87, no. 2, pp. 271–284, May 2005, doi: 10.1162/0034653053970339

[83] Raschky P. A., ‘Institutions and the losses from natural disasters’, *Nat*. *Hazards Earth Syst*. *Sci*., vol. 8, no. 4, pp. 627–634, Jul. 2008, doi: 10.5194/nhess-8-627-2008

[84] Anderson R. M., Heesterbeek H., Klinkenberg D., and Hollingsworth T. D., ‘How will country-based mitigation measures influence the course of the COVID-19 epidemic?’, *The Lancet*, vol. 395, no. 10228, pp. 931–934, Mar. 2020, doi: 10.1016/S0140-6736(20)30567-5 32164834 PMC7158572

[85] van der Weerd W., Timmermans D. R., Beaujean D. J., Oudhoff J., and van Steenbergen J. E., ‘Monitoring the level of government trust, risk perception and intention of the general public to adopt protective measures during the influenza A (H1N1) pandemic in the Netherlands’, *BMC Public Health*, vol. 11, no. 1, p. 575, Dec. 2011, doi: 10.1186/1471-2458-11-575 21771296 PMC3152536

[86] Chisadza C., Clance M., and Gupta R., ‘Government Effectiveness and the COVID-19 Pandemic’, *Sustainability*, vol. 13, no. 6, p. 3042, Mar. 2021, doi: 10.3390/su13063042

[87] Petrović D., Petrović M., Bojković N., and Čokić V. P., ‘An integrated view on society readiness and initial reaction to COVID–19: A study across European countries’, *PLOS ONE*, vol. 15, no. 11, p. e0242838, Nov. 2020, doi: 10.1371/journal.pone.0242838 33227029 PMC7682891

[88] Bargain O. and Aminjonov U., ‘Trust and compliance to public health policies in times of COVID-19’, *J*. *Public Econ*., vol. 192, p. 104316, Dec. 2020, doi: 10.1016/j.jpubeco.2020.104316 33162621 PMC7598751

[89] Verger P. and Peretti-Watel P., ‘Understanding the determinants of acceptance of COVID-19 vaccines: a challenge in a fast-moving situation’, *Lancet Public Health*, vol. 6, no. 4, pp. e195–e196, Apr. 2021, doi: 10.1016/S2468-2667(21)00029-3 33556329 PMC7864794

[90] Soveri A., Karlsson L. C., Antfolk J., Lindfelt M., and Lewandowsky S., ‘Unwillingness to engage in behaviors that protect against COVID-19: the role of conspiracy beliefs, trust, and endorsement of complementary and alternative medicine’, *BMC Public Health*, vol. 21, no. 1, p. 684, Dec. 2021, doi: 10.1186/s12889-021-10643-w 33832446 PMC8027965

[91] European Observatory on Health Systems and Policies, Thomas S, Sagan A, Larkin J, Cylus J, and et al., ‘Strengthening health systems resilience: key concepts and strategies.’, in Health Systems and Policy Analysis; Policy brief 36. Copenhagen: World Health Organization. Regional Office for Europe., 2020, p. 29p. [Online]. Available: https://apps.who.int/iris/handle/10665/332441 32716618

[92] González-Padilla D. A. and Tortolero-Blanco L., ‘Social media influence in the COVID-19 Pandemic’, *Int*. *Braz*. *J*. *Urol*., vol. 46, no. suppl 1, pp. 120–124, Jul. 2020, doi: 10.1590/S1677-5538.IBJU.2020.S121 32550706 PMC7719982

[93] Chan A. K. M., Nickson C. P., Rudolph J. W., Lee A., and Joynt G. M., ‘Social media for rapid knowledge dissemination: early experience from the COVID-19 pandemic’, *Anaesthesia*, vol. 75, no. 12, pp. 1579–1582, Dec. 2020, doi: 10.1111/anae.15057 32227594 PMC7228334

[94] Dirani K. M. et al., ‘Leadership competencies and the essential role of human resource development in times of crisis: a response to Covid-19 pandemic’, *Hum*. *Resour*. *Dev*. *Int*., vol. 23, no. 4, pp. 380–394, Aug. 2020, doi: 10.1080/13678868.2020.1780078

[95] Chen Y., kumara E. K., and Sivakumar V., ‘Investigation of finance industry on risk awareness model and digital economic growth’, *Ann*. *Oper*. *Res*., Oct. 2021, doi: 10.1007/s10479-021-04287-7

[96] Gao H., Shi D., and Zhao B., ‘Does good luck make people overconfident? Evidence from a natural experiment in the stock market’, *J*. *Corp*. *Finance*, vol. 68, p. 101933, Jun. 2021, doi: 10.1016/j.jcorpfin.2021.101933

[97] Pickett K. E. and Wilkinson R. G., ‘Income inequality and health: A causal review’, *Soc*. *Sci*. *Med*., vol. 128, pp. 316–326, Mar. 2015, doi: 10.1016/j.socscimed.2014.12.031 25577953

[98] Ahmed F., Ahmed N., Pissarides C., and Stiglitz J., ‘Why inequality could spread COVID-19’, *Lancet Public Health*, vol. 5, no. 5, p. e240, May 2020, doi: 10.1016/S2468-2667(20)30085-2 32247329 PMC7270465

[99] Takian A., Kiani M. M., and Khanjankhani K., ‘COVID-19 and the need to prioritize health equity and social determinants of health’, *Int*. *J*. *Public Health*, vol. 65, no. 5, pp. 521–523, Jun. 2020, doi: 10.1007/s00038-020-01398-z 32462311 PMC8825633

[100] Iboi E. et al., ‘Impact of Public Health Education Program on the Novel Coronavirus Outbreak in the United States’, *Front*. *Public Health*, vol. 9, p. 630974, Mar. 2021, doi: 10.3389/fpubh.2021.630974 33791268 PMC8005517

[101] Czech K., Davy A., and Wielechowski M., ‘Does the COVID-19 Pandemic Change Human Mobility Equally Worldwide? Cross-Country Cluster Analysis’, *Economies*, vol. 9, no. 4, p. 182, Nov. 2021, doi: 10.3390/economies9040182

[102] Dzator J. et al., ‘Policy Stringency, Handwashing and COVID-19 cases: Evidence from Global dataset’, *Health Policy Technol*., p. 100574, Nov. 2021, doi: 10.1016/j.hlpt.2021.100574 34786329 PMC8574075

[103] Ramalingam B. and Prabhu J., ‘Innovation, development and COVID-19: Challenges, opportunities and ways forward’, OECD Policy Responses to Coronavirus (COVID-19), Dec. 2020. Accessed: Jun. 27, 2022. [Online]. Available: https://www.oecd-ilibrary.org/development/innovation-development-and-covid-19-challenges-opportunities-and-ways-forward_0c976158-en

[104] Caballero-Morales S.-O., ‘Innovation as recovery strategy for SMEs in emerging economies during the COVID-19 pandemic’, *Res*. *Int*. *Bus*. *Finance*, vol. 57, p. 101396, Oct. 2021, doi: 10.1016/j.ribaf.2021.101396 33558782 PMC7857984

[105] Lv Z., Chen D., and Lv H., ‘Smart City Construction and Management by Digital Twins and BIM Big Data in COVID-19 Scenario’, *ACM Trans*. *Multimed*. *Comput*. *Commun*. *Appl*., p. 3529395, Apr. 2022, doi: 10.1145/3529395

[106] Chen D., Gao H., and Ma Y., ‘Human Capital-Driven Acquisition: Evidence from the Inevitable Disclosure Doctrine’, *Manag*. *Sci*., vol. 67, no. 8, pp. 4643–4664, Aug. 2021, doi: 10.1287/mnsc.2020.3707

[107] Wu Y. and Zhu W., ‘The Role of CSR Engagement in Customer-Company Identification and Behavioral Intention During the COVID-19 Pandemic’, *Front*. *Psychol*., vol. 12, p. 721410, Aug. 2021, doi: 10.3389/fpsyg.2021.721410 34475843 PMC8407001

[108] Schakel H. C., Wu E. H., and Jeurissen P., ‘Fiscal rules, powerful levers for controlling the health budget? Evidence from 32 OECD countries’, *BMC Public Health*, vol. 18, no. 1, p. 300, Dec. 2018, doi: 10.1186/s12889-018-5198-y 29490651 PMC5831227

[109] WHO, ‘COVID-19 Research and Innovation Powering the world’s pandemic response–now and in the future’, World Health Organization, Geneva, 2022. [Online]. Available: https://cdn.who.int/media/docs/default-source/blue-print/achievement-report-_grif_web_finalversion15.pdf?sfvrsn=39052c73_9&download=true

[110] van der Voorn T., van den Berg C., Quist J., and Kok K., ‘Making waves in resilience: Drawing lessons from the COVID-19 pandemic for advancing sustainable development’, *Curr*. *Res*. *Environ*. *Sustain*., vol. 4, p. 100171, 2022, doi: 10.1016/j.crsust.2022.100171 35720270 PMC9189097

[111] Aboelnaga S., Tóth T., and Neszmélyi G. I., ‘Calculations on Ecological Footprint as a tool for land use planning and development on V4 countries’, *DETUROPE—Cent*. *Eur*. *J*. *Tour*. *Reg*. *Dev*., vol. 13, no. 1, pp. 24–38, Jul. 2021, doi: 10.32725/det.2021.002

[112] Noy I., Doan V. N., Ferrarini B., and Park D., ‘Measuring the Economic Risk of Epidemics’, *SSRN Electron*. *J*., 2019, doi: 10.2139/ssrn.3518964

[113] Diop S., Asongu S. A., and Nnanna J., ‘COVID‐19 economic vulnerability and resilience indexes: Global evidence’, *Int*. *Soc*. *Sci*. *J*., vol. 71, no. S1, pp. 37–50, Nov. 2021, doi: 10.1111/issj.12276 34548690 PMC8447304

[114] Marti L. and Puertas R., ‘European countries’ vulnerability to COVID-19: multicriteria decision-making techniques’, *Econ*. *Res*.*-Ekon*. *Istraživanja*, vol. 34, no. 1, pp. 3309–3320, Jan. 2021, doi: 10.1080/1331677X.2021.1874462

[115] Rai P. K., Sonne C., Song H., and Kim K.-H., ‘The effects of COVID-19 transmission on environmental sustainability and human health: Paving the way to ensure its sustainable management’, *Sci*. *Total Environ*., vol. 838, p. 156039, Sep. 2022, doi: 10.1016/j.scitotenv.2022.156039 35595144 PMC9113776

[116] Lekagul A., Chattong A., Rueangsom P., Waleewong O., and Tangcharoensathien V., ‘Multi-dimensional impacts of Coronavirus disease 2019 pandemic on Sustainable Development Goal achievement’, *Glob*. *Health*, vol. 18, no. 1, p. 65, Dec. 2022, doi: 10.1186/s12992-022-00861-1 35761400 PMC9235167

[117] Wang Q. and Huang R., ‘The impact of COVID-19 pandemic on sustainable development goals–A survey’, *Environ*. *Res*., vol. 202, p. 111637, Nov. 2021, doi: 10.1016/j.envres.2021.111637 34233155 PMC8566018

[118] D’Adamo I., Gastaldi M., and Morone P., ‘Economic sustainable development goals: Assessments and perspectives in Europe’, *J*. *Clean*. *Prod*., vol. 354, p. 131730, Jun. 2022, doi: 10.1016/j.jclepro.2022.131730

[119] Resce G. and Schiltz F., ‘Sustainable Development in Europe: A Multicriteria Decision Analysis’, *Rev*. *Income Wealth*, vol. 67, no. 2, pp. 509–529, Jun. 2021, doi: 10.1111/roiw.12475

[120] Ricciolini E. et al., ‘Assessing Progress Towards SDGs Implementation Using Multiple Reference Point Based Multicriteria Methods: The Case Study of the European Countries’, *Soc*. *Indic*. *Res*., vol. 162, no. 3, pp. 1233–1260, Aug. 2022, doi: 10.1007/s11205-022-02886-w 35125614 PMC8802750

[121] Ranjbari M. et al., ‘Three pillars of sustainability in the wake of COVID-19: A systematic review and future research agenda for sustainable development’, *J*. *Clean*. *Prod*., vol. 297, p. 126660, May 2021, doi: 10.1016/j.jclepro.2021.126660 34785869 PMC8580193

[122] Google, ‘COVID-19 Community Mobility Reports’, Google, 2021. https://www.google.com/covid19/mobility/

[123] Hale T., Webster S., Petherick A., Phillips T., and Kira B., ‘Oxford COVID-19 Government Response Tracker. Blavatnik School of Government’, 2020. www.bsg.ox.ac.uk/covidtracker (accessed Jul. 20, 2020).

[124] Pollesch N. L. and Dale V. H., ‘Normalization in sustainability assessment: Methods and implications’, *Ecol*. *Econ*., vol. 130, pp. 195–208, Oct. 2016, doi: 10.1016/j.ecolecon.2016.06.018

[125] OECD, Ed., *Handbook on constructing composite indicators*: *methodology and user guide*. Paris: OECD, 2008.

[126] Mazziotta M. and Pareto A., ‘Methods For Constructing Composite Indices: One For All Or All For One?’, *Riv*. *Ital*. *Econ*. *Demogr*. *E Stat*., vol. 67, no. 2, pp. 67–80, 2013.

[127] Cheng I.-H., Kirilenko A., and Xiong W., ‘Convective Risk Flows in Commodity Futures Markets*’, *Rev*. *Finance*, vol. 19, no. 5, pp. 1733–1781, Aug. 2015, doi: 10.1093/rof/rfu043

[128] Kruskal W. H., ‘A Nonparametric test for the Several Sample Problem’, *Ann*. *Math*. *Stat*., vol. 23, no. 4, pp. 525–540, Dec. 1952, doi: 10.1214/aoms/1177729332

[129] Kruskal W. H. and Wallis W. A., ‘Use of Ranks in One-Criterion Variance Analysis’, *J*. *Am*. *Stat*. *Assoc*., vol. 47, no. 260, pp. 583–621, Dec. 1952, doi: 10.1080/01621459.1952.10483441

[130] Wilcoxon F., ‘Individual Comparisons by Ranking Methods’, in *Breakthroughs in Statistics*, Kotz S.and Johnson N. L., Eds., in Springer Series in Statistics. New York, NY: Springer New York, 1992, pp. 196–202. doi: 10.1007/978-1-4612-4380-9_16

[131] Benjamini Y. and Hochberg Y., ‘Controlling the False Discovery Rate: A Practical and Powerful Approach to Multiple Testing’, *J*. *R*. *Stat*. *Soc*. *Ser*. *B Methodol*., vol. 57, no. 1, pp. 289–300, Jan. 1995, doi: 10.1111/j.2517-6161.1995.tb02031.x

