# Supplementary figures and images for: COVID-19 resilience index in European Union countries based on their risk and readiness scale
Source: PLoS One. 2023 Aug 4;18(8):e0289615. doi: 10.1371/journal.pone.0289615 (PMC10403121; doi:10.1371/journal.pone.0289615)

**Appendix A:**

Risk


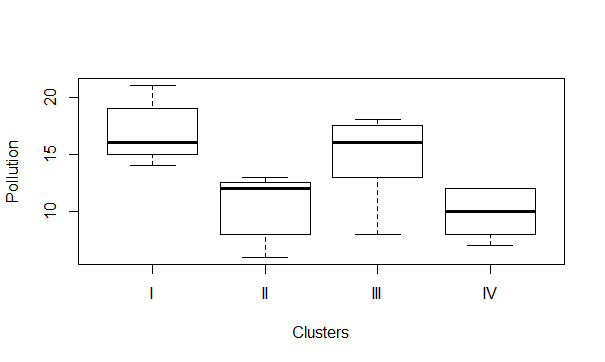

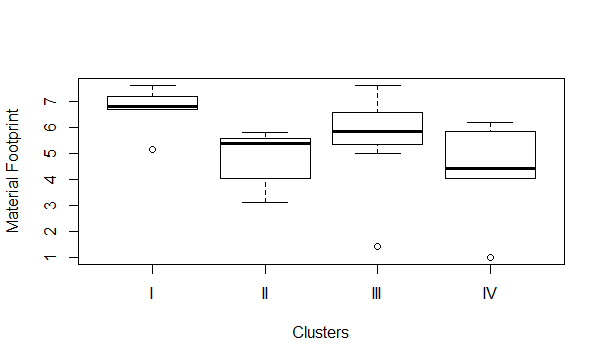


Readiness


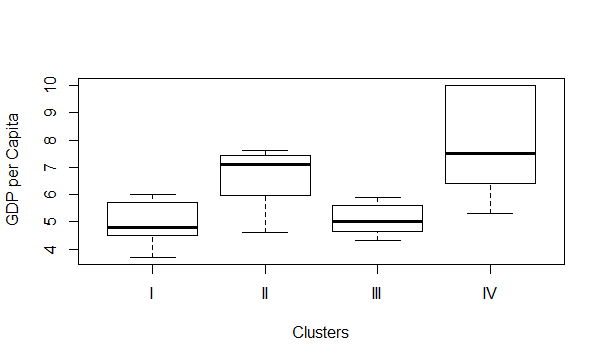

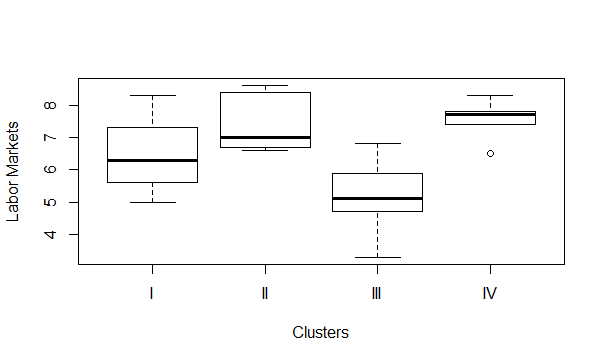


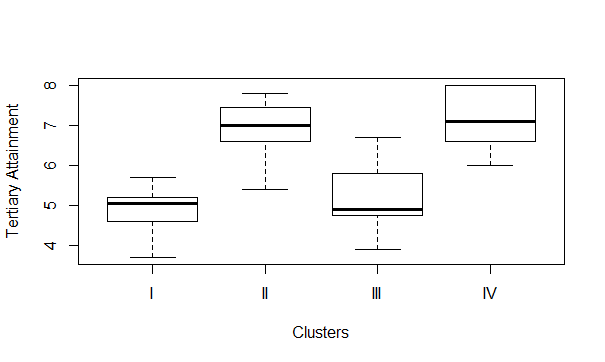

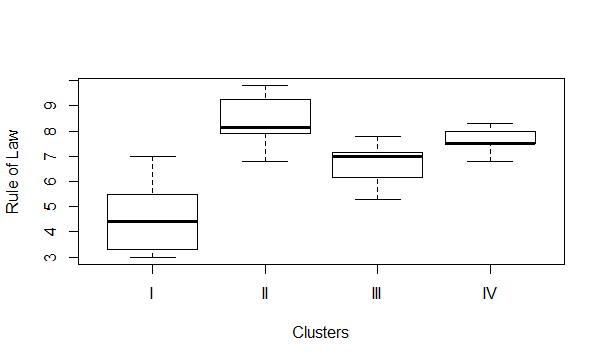


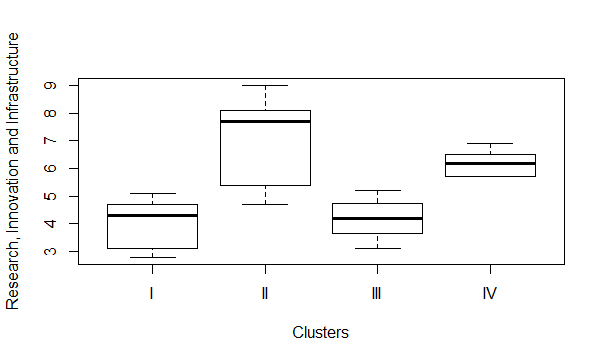

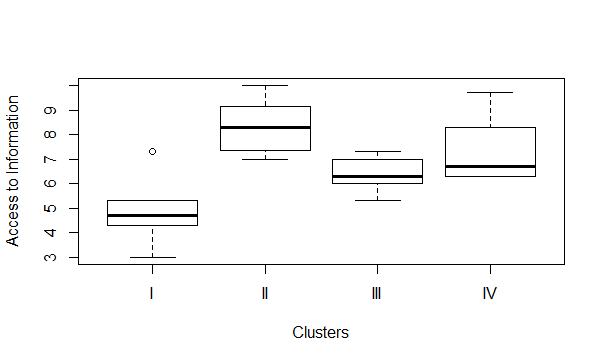


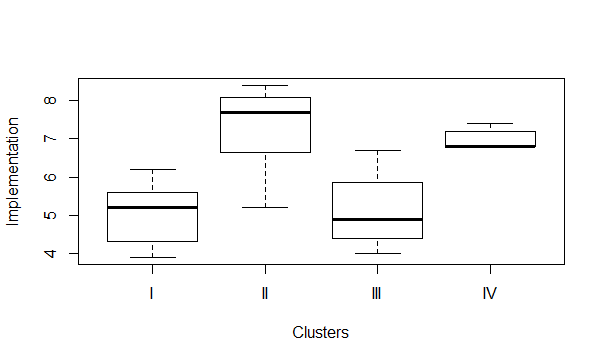

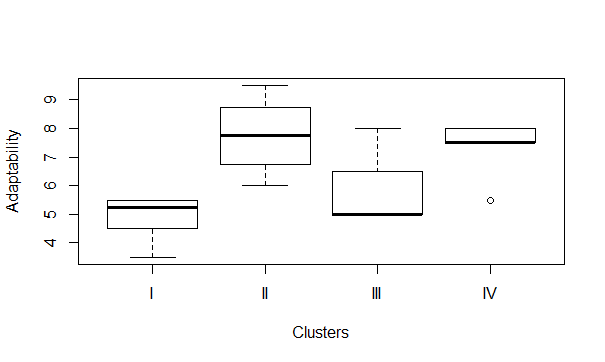

Supplement: S1 Appendix — (DOCX) [file pone.0289615.s001.docx]
